# Arteria: An automation system for a sequencing core facility

**DOI:** 10.1093/gigascience/giz135

**Published:** 2019-12-11

**Authors:** Johan Dahlberg, Johan Hermansson, Steinar Sturlaugsson, Mariya Lysenkova, Patrik Smeds, Claes Ladenvall, Roman Valls Guimera, Florian Reisinger, Oliver Hofmann, Pontus Larsson

**Affiliations:** 1 Department of Medical Sciences, Molecular Medicine and Science for Life Laboratory, Uppsala University, Box 1432, BMC 751 44, Uppsala, Sweden; 2 Department of Immunology, Genetics and Pathology and Science for Life Laboratory, Uppsala University, Rudbecklaboratoriet, 751 84, Uppsala, Sweden; 3 University of Melbourne Center for Cancer Research, University of Melbourne, Victorian Comprehensive Cancer Centre, Level 10, UMCCR, 305 Grattan St, Melbourne VIC 3000, Australia

**Keywords:** automation, sequencing, orchestration, workflows

## Abstract

**Background:**

In recent years, nucleotide sequencing has become increasingly instrumental in both research and clinical settings. This has led to an explosive growth in sequencing data produced worldwide. As the amount of data increases, so does the need for automated solutions for data processing and analysis. The concept of workflows has gained favour in the bioinformatics community, but there is little in the scientific literature describing end-to-end automation systems. Arteria is an automation system that aims at providing a solution to the data-related operational challenges that face sequencing core facilities.

**Findings:**

Arteria is built on existing open source technologies, with a modular design allowing for a community-driven effort to create plug-and-play micro-services. In this article we describe the system, elaborate on the underlying conceptual framework, and present an example implementation. Arteria can be reduced to 3 conceptual levels: orchestration (using an event-based model of automation), process (the steps involved in processing sequencing data, modelled as workflows), and execution (using a series of RESTful micro-services). This creates a system that is both flexible and scalable. Arteria-based systems have been successfully deployed at 3 sequencing core facilities. The Arteria Project code, written largely in Python, is available as open source software, and more information can be found at https://arteria-project.github.io/ .

**Conclusions:**

We describe the Arteria system and the underlying conceptual framework, demonstrating how this model can be used to automate data handling and analysis in the context of a sequencing core facility.

## Findings

### Challenges in, and approaches to, processing sequencing data

Nucleotide sequencing is the practice of determining the order of bases of the nucleic acid sequences that form the foundation of all known forms of life. It has been hugely successful as a research tool, used to understand basic biology [1–3], and is also applied as a tool for precision medicine [4]. Major technological advances during the past decade have enabled high-throughput approaches for massively parallel sequencing (MPS) [5]. The amount of data generated globally from MPS has boomed in recent years and has been projected to reach a yearly production of 10^21^ bp per year by 2025, demanding 2–40 exabytes (10^18^) per year of storage [6]. This massive expansion places new demands on how data are analysed, stored, and distributed.

Much of this nucleotide sequencing is carried out at sequencing core facilities, which perform sequencing as a service. The kinds of services provided vary widely, but most facilities provide at least some processing of raw sequencing data, typically conversion to a standard FASTQ format at a minimum [7]. More advanced bioinformatic analysis may involve passing the data through a pipeline of software processes. Such processes often require manual initiation or intervention, creating a significant overhead of labor and increased turnaround times.

Automation of both laboratory and computational procedures is crucial in order for a sequencing facility to scale the number of samples processed. Furthermore, automated processes reduce the risk of human errors, which contributes to higher quality data.

However, there are challenges to automating these processes. Despite the high standardization of laboratory protocols, a number of factors create a combinatorial situation that makes every laboratory unique, including small variation in procedures, infrastructure, and surrounding systems. This requires the development of bespoke solutions to manage specific situations. One example of these custom solutions is laboratory information management systems (LIMS), which are used to track the laboratory procedures that a sample is subjected to, and to perform surrounding utility tasks such as generating instruction files for pipetting robots. LIMS are often based on extensible platforms in which specific laboratory protocols can be implemented.

The potential complexity in the laboratory will often extend into the computational environment. The specific nature of the infrastructure and services offered places wide-ranging demands on the computational systems developed to support management and high-throughput analysis of sequencing data. This has led most sequencing core facilities to develop their own custom solutions to this problem, and these are often highly coupled to the infrastructure and process of that particular core facility [8].

These systems need to be designed not only to support the analysis of data but to address additional aspects associated with operating a sequencing facility. Examples include automatically starting data processing when a sequencing run has finished, archiving of data to remote storage, and selective data removal. These *operational* aspects have not been thoroughly investigated in the scientific literature but are essential when taking a bird's-eye view of the complete process of refining raw MPS data to scientific results on a high-throughput scale. Tackling these issues involves examination of how higher level orchestration, integration, and management of workflows can be done in an efficient yet flexible manner, while providing a clear enough understanding of the system so that changes can be implemented with minimal mental overhead and risk of breaking existing functionality. Arteria fills a niche by providing a systematic way of approaching the operational aspects of data management and analysis of MPS data.

In recent years there has been an increased interest in workflow systems, both in academia [8–11] and in industry [12, 13]. Typically, these systems model a workflow as a directed acyclic graph of dependencies between computational tasks. The core concepts that define workflows, as used here, are defined in Table 1.

**Table 1:** Definitions

Term	Description
Action	A computational unit of work, e.g., processing a file or inserting data into a database. This is sometimes referred to as a task.
Process	A set of steps that have to be finished to achieve a particular goal, e.g., delivering data to a user. A process can include automated and manual steps.
Workflow	A workflow models a process, as a number of "actions" following each other. This can be described by a directed acyclic graph.

These workflows are often designed to be executed on a per-project or per-sample level, with parameters being provided manually by the operator. Furthermore, they typically focus on tying together command line programs that have files as inputs and outputs. This model is well suited for processing large amounts of data, where all samples in a project can be analysed the same way. However, for institutions that provide sequencing as a service to multiple users or projects, this type of system does not scale well, due to the need for manual intervention at different stages of the process. Additionally, many sequencing core facilities will have workflows where file input/output is not the most natural solution. For example, updating a database or emailing reports will not generate files by default. Thus there is a need for systems to address these challenges, which can be thought of as operational rather than analytical.

One example of a system addressing the operational challenges outlined above in the context of a sequencing core facility is described by Cuccuru et al. [14]. They describe a system with a central automator that handles orchestration of the processes in an event-based manner, using the Galaxy platform [15] as a separate workflow manager. The Galaxy platform provides a web-based interface, making bioinformatic analysis accessible to users who lack the training to use command line tools. The system's automator is based on daemons monitoring a RabbitMQ [16] based event queue. While this system shares ideas with the Arteria system, it does not have the same focus on decoupling the system from the implementation at the facility in question. Furthermore, the Arteria system benefits from building on top of existing industry standards for event-based automation systems rather than building these from scratch.

Herein, we describe the automation system Arteria, which is available as open source software at GitHub [17]. Arteria uses the open source automation platform StackStorm [18] for event-based orchestration, the Mistral [19] workflow engine for process modelling, and Python micro-services for action execution. Arteria has been successfully implemented at 3 separate sequencing core facilities to date: the SNP&SEQ Technology Platform at Science For Life Laboratory, Clinical Genomics Uppsala at Science For Life Laboratory, and the University of Melbourne Center for Cancer Research.

### System overview

The Arteria system is built with 2 existing open source technologies at its core: the StackStorm automation platform [18] and the Mistral workflow service [19]. By adopting existing open source solutions and extending them for our domain, we are able to leverage the power of a larger open source community. This has allowed us to focus on our specific use case: automation of sequencing data processing.

**Figure 1:**
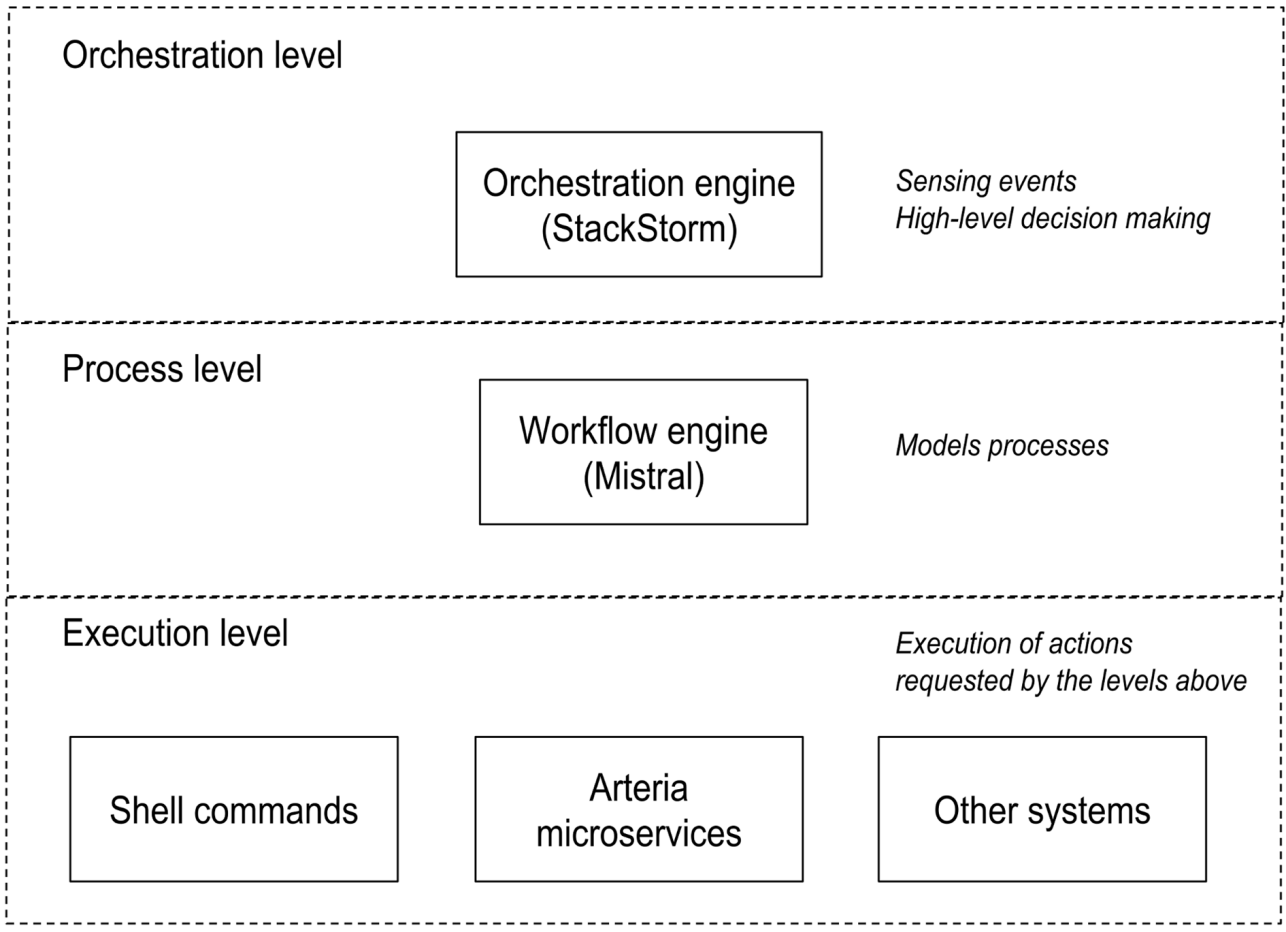
An overview of the conceptual levels of the Arteria project.

The Arteria system can be divided into 3 conceptual levels, a model that has been adopted from StackStorm: the orchestration level, the process level, and the execution level (Fig. 1). For an overview of how information flows between levels, see Supplementary Fig. 1.

At the highest level, the orchestration level, StackStorm serves as the central point of automation. It provides both command line and web interfaces through which an operator can interact with the system. It uses an event-based model to decide when actions should be triggered. For example, the completion of a sequencing run is an event that may trigger further actions to be taken by the system.

At the process level, internal processes are modelled as workflows using the Mistral service. For example, a workflow triggered by the completion of a sequencing run may then carry out basic processing, gather quality control data, and transfer the data to a high-performance computing (HPC) resource.

Finally, at the execution level, actions are carried out. This level includes multiple modes of execution, ranging from a shell command on a local or remote machine, to interaction with surrounding systems such as a LIMS, to invoking a micro-service. The final mode, the micro-service, is the one favoured by Arteria. The advantages of the micro-service approach include system flexibility and the decoupling of implementation details of the execution from the process that invokes it.

This separation of the system into levels makes the Arteria system easier to deconstruct, and places implementation details at the correct level of abstraction. In addition, Arteria enforces a separation of concerns that makes it easier to update or replace individual components without having to make large changes to the system as a whole. This creates a flexible system that is able to meet the scaling demands placed on sequencing core facilities, where protocols are modified and new instrumentation is routinely implemented.

#### Event-based orchestration

**Figure 2:**
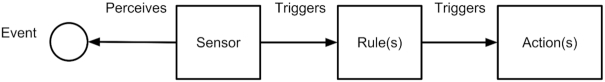
Description of the StackStorm event model. Sensors will perceive events in the environments, e.g., a file being created or a certain time of day occuring. This passes information to the rule layer where the data are evaluated and depending on which, if any, criteria are fulfilled 1 or more actions are triggered. Actions can be single commands or full workflows to be executed.

At the orchestration level, Arteria uses StackStorm to coordinate tasks. A core concept of StackStorm is its event-based model of automation (see Fig. 2). It uses sensors to detect events from the environment. A typical example is a sequencing instrument finishing a run. The event parameters are then passed through a rule layer that decides which, if any, action or workflow should be started. If an action or workflow should be started, sufficient parameters need to be passed on by the rule layer. This simple yet powerful abstraction makes the Arteria system and its behaviour simple to understand. In addition to triggering actions in response to sensor events, an operator can manually initiate an action via a command line or web interface. When manually initiating an action, the operator must provide the parameters necessary to start the action, as well as any other optional parameters to modify the action's default behaviour.

Furthermore, StackStorm provides per-action monitoring capabilities. Each action taken by the Arteria system is assigned a unique ID, allowing operators to follow the progress of processes in the system. An additional advantage is the ability to create audit trails, which are both useful internally and required to accredit systems; e.g., it is required by the European quality standard ISO/IEC 17025 [20] under which the SNP&SEQ Technology Platform operates. Finally, providing a centralized interface to the underlying processes means that operators require less knowledge of the underlying components and direct access to fewer systems, which is an advantage from a security perspective.

#### Modelling processes as workflows

At the process level, the process of a particular use case is modelled using the Mistral workflow language. Mistral uses a declarative yaml syntax to define a workflow, which allows for the definition of complicated dependency structures. It supports the use of conditionals, forking (defining multiple tasks that must be run after the completion of a given task), and joining (the synchronization of multiple parallel workflow branches and aggregation of their data). It will execute actions that do not have dependencies on each other concurrently. This simple and powerful syntax has the additional advantage of serving as a human-readable documentation of the modelled process. Modelling a process as a workflow can mean formalizing the documentation of an existing process into a Mistral workflow, thus reducing the amount of manual work required as well as reducing the risk of human errors.

#### Micro-services provide a flexible execution model

Finally, at the execution level, any action that needs to be carried out by the system is performed. Arteria favours the use of single-purpose micro-service executors, and these provide the actual functionality and logic for performing the actions. These micro-services are invoked from the process level through an HTTP API, making the communication simple and allowing for easy integration with other services. An example of such a micro-service is the one provided by Arteria to run the preprocessing program Illumina bcl2fastq [21], which processes the raw data produced by an Illumina sequencing instrument and converts it to the industry standard FASTQ format. However, a micro-service is not required; the Arteria approach is flexible enough that it supports running a shell command or invoking another service, e.g., a LIMS.

Using micro-services as the primary execution mode increases the flexibility of the Arteria system as the implementation details of *how* something is run are decoupled from *when* it is run. Furthermore, such micro-services can be reused across systems, or even centers, creating an avenue for reuse and collaboration, which sets the Arteria approach apart from other sequencing core facility systems that are typically tightly coupled to the process and infrastructure of the sequencing core facility that developed them.

Finally, decoupling the execution layer has allowed us to build simple interfaces for existing software, thus significantly reducing the burden of having to re-implement components that have been used reliably for a long time in operation.

### Implementation

Arteria is made up of publicly available software packages, written largely in Python, that can be grouped into 2 components. The first component is arteria-packs, a plugin for the orchestration engine Stackstorm, which acts as a starting template for individual core facilities to build their own implementation.

The second component is a series of single-responsibility REST micro-services, made up of both general-interest packages that can be reused across sequencing core facilities and tailor-made services that cater to a single facility's specific needs. These provide specific functionality, such as running the Illumina bcl2fastq program, checking whether a runfolder is ready to be analysed, or removing data once certain criteria are met. These micro-services, and others, are available from GitHub [17].

The package arteria-packs is available for download at GitHub [22] (the accompanying README provides detailed installation instructions). The purpose of this package is two-fold: first, to act as a demonstration illustrating a minimal but complete Arteria system; second, to serve as a template for sequencing core facilities to build upon in developing their own Arteria implementations.

During the set-up process for arteria-packs, Docker is used to create an environment that comprises Stackstorm, its dependencies, and 3 general-interest Arteria micro-services: arteria-runfolder, arteria-bcl2fastq, and checkQC.

The repository provides the sample units detailed in Table 2.

**Table 2:** Descriptions of concepts in arteria-packs sample implementation

Concept	Definition	arteria-packs implementation
Actions	Encapsulate system tasks	Micro-services arteria-runfolder, arteria-bcl2fastq, and checkQC
Workflows	Tie actions together	Mistral workflow defined in workflow_bcl2fastq_and_checkqc.yaml
Sensors	Pick up events from the environment	RunfolderSensor defined in runfolder_sensor.yaml, which detects runfolders ready for processing
Rules	Parse events from sensors and determine whether an action or a workflow should be initiated	Defined in when_runfolder_is_ready_start_bcl2fastq.yaml; fires bcl2fastq workflow when a runfolder is ready

The workflow, defined in workflows/bclfastq_and_checkqc.yaml, outlines the following actions to detect and process a runfolder:

- get_runfolder_name
- mark_as_started
- start_bcl2fastq
- poll_bcl2fastq
- checkqc
- mark_as_done
- mark_as_error

In arteria-packs, the example workflow operates as follows. The *runfolder_sensor* routinely polls the arteria-runfolder service to retrieve information about unprocessed runfolders. When the service returns the *runfolder_ready* event, the Stackstorm sensor rule *when_runfolder_is_ready_start_bcl2fastq* is triggered, initiating arteria-bcl2fastq, which demultiplexes data and converts the binary base call (BCL) format to FASTQ. The Stackstorm instance will poll arteria-bcl2fastq until it receives a “done” status. Following this, a set of quality criterias are checked against the data using checkqc. The arteria-runfolder service is then invoked to mark the runfolder with the state of “done” or “error.”

To test the workflow, a runfolder containing Illumina-generated sequencing data may be placed in the "docker-mountpoints/monitored-folder" directory (see sample runfolder [23]).

A command can then be run to initiate the workflow manually. Alternatively, the rule *when_runfolder_is_ready_start_bcl2fastq* can be enabled, allowing automatic processing of any ready runfolder. Refer to the repository README for details.

The arteria-packs repository serves as a starting point for a sequencing core facility to implement its own actions, workflows, sensors, and rules.

### Deployment scenario and usage statistics

#### SNP&SEQ Technology Platform

The SNP&SEQ Technology Platform sequencing core facility at Science for Life Laboratory provides sequencing and genotyping as a service to the Swedish research community. Projects from a wide variety of fields are accepted, ranging from clinical research projects to environmental sciences. In addition, the facility provides a large number of assays; some examples are DNA, RNA, and bisulfite-converted library preparations and sequencing, as well as the sequencing of libraries prepared by users.

**Figure 3:**
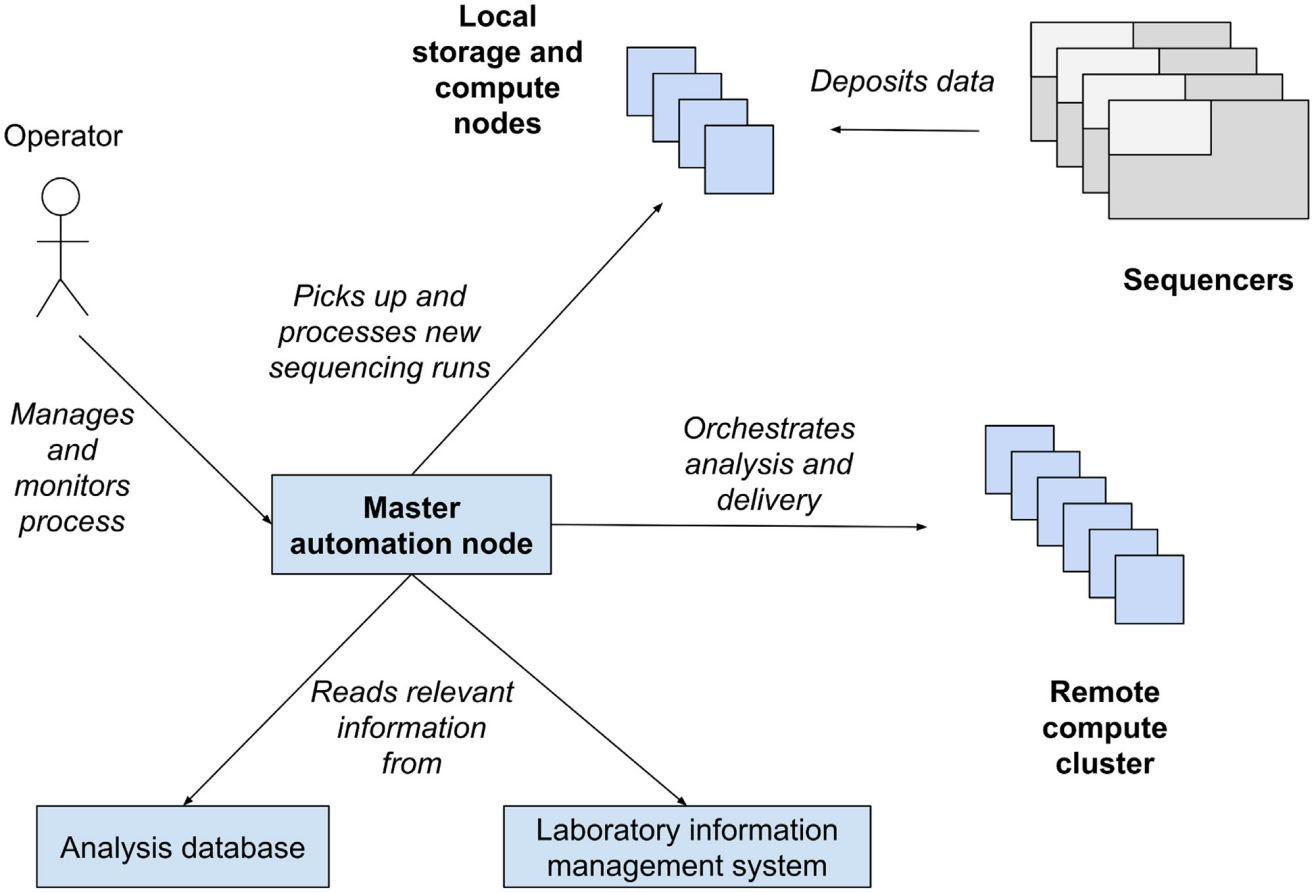
Schematic view of a system deployment scenario, showing how data are written to the local storage and compute nodes from the sequencing machines, and how the system uses information and resources from multiple sources to coordinate the process. The operator can then monitor and control the processes from the single interface provided at the master automation node.

At the SNP&SEQ Technology Platform, the Arteria system is deployed in a distributed environment (see Fig. 3) and orchestrates actions across a local cluster of 10 nodes used for storage and preliminary analysis with 208 cores and 120 TB of storage capacity, as well as an HPC cluster at the Uppsala Multidisciplinary Center for Advanced Computational Science (UPPMAX) HPC center with 4,000 cores and 2.1 PB storage. This system is fully capable of supporting the fleet of 8 Illumina sequencers (2 NovaSeq, 3 HiSeqX, 1 HiSeq 2500, 1 MiSeq, and 1 iSeq) that are currently in use at the SNP&SEQ Technology Platform.

The SNP&SEQ Technology platform uses Arteria workflows that automatically pick up data as the sequencing instrument finishes a sequencing run, convert it into the industry standard FASTQ format, check it against a set of quality criteria, upload data to off-site archiving, and transfer it to the HPC cluster. Other workflows include data delivery to users on a per-run or per-project basis, synchronizing data between local and remote systems, weekly report generation, and automatic read-back tests of archived sequencing data.

Adopting Arteria at the SNP&SEQ Technology platform has reduced the amount of manual work required to process sequencing data. It has also provided a convenient interface through which most of our internal processes can now be monitored. Furthermore, the detailed process and error logs provided by Arteria are used to build reporting dashboards, allowing staff to see which processes are bottlenecks and to set concrete, quantitative automation goals.

Since being deployed at the SNP&SEQ Technology Platform, the Arteria system has been used to process >50,000 samples and 715 projects, which corresponds to ∼1,213 terabases of sequencing data.

#### Clinical Genomics, Uppsala

At the Clinical Genomics Uppsala (Science for Life Laboratory) MPS analyses are developed and implemented for clinical use with the aim to improve diagnostics and allow for targeted treatments. The turnaround time for certain samples must be short, preferably 1–2 hours from when the facility obtains raw data to when processed data are sent to clinical staff for manual interpretation and reporting.

Raw sequence data are produced at the Uppsala University Hospital, but at present the hospital lacks the capacity to analyse MPS data within the required time. To overcome this, the bioinformatic processing is performed on a private cluster with 8 nodes at Uppsala University. Because of this set-up our analyses include transfers between networks, including the more secure hospital network that only allows for communication to be initiated from within the hospital.

Our Arteria implementation is deployed in a Docker environment and consists of Arteria and in-house–developed workflows. It is routinely used to push data from the hospital to our cluster located in the Uppsala University Network. When raw data are transferred to the cluster, Arteria subsequently initiates analyses on the cluster, pulls back results into the hospital network, and archives data. We also use Arteria to send out emails when results are available or when an error occurs in the sample processing.

The need for a minimal turnaround time and our network limitations makes Arteria and its micro-services a key component for us. The solution automates several tasks that previously were done manually, reducing labour-intensive and time-consuming repetitive steps in our pipelines. After being deployed, the system has processed 2,176 samples originating from 362 sequence runs. Apart from automating a repetitive task, we estimate that we now save ∼2 hours of bioinformatician working time for each sequencing run and slightly improved our turnaround time.

We are currently working on implementing the system on remaining pipelines that we have set up. In the near future we will also implement a solution for the automatic conversion of raw sequence data from sequencing machines to the standard FASTQ format, using the arteria-bcl2fastq micro-service.

#### University of Melbourne Center for Cancer Research

The University of Melbourne Center for Cancer Research (UMCCR) aims to improve cancer patient outcome and enable personalized medicine through rapid whole-genome sequencing (WGS) and whole-transcriptome sequencing (WTS) of tumour samples. Operating in a clinical, accredited environment requires reproducible and traceable data management, which the Center implements through Arteria, providing crucial automation, error notifications, provenance, LIMS control, centralized monitoring, and orchestration.

Rapid WGS/WTS data are generated by Illumina's NovaSeq platform and processed through the bcbio framework [24] at both a commercial cloud provider (Amazon Web Services) and traditional HPC centers within Australia. Arteria automates primary data movement between computational environments and handles distribution of results to a cBio portal, long-term archival storage facilities, and other downstream services such as automatic report generation through the Personal Cancer Genome Reporter [25]. This software suite includes multi-gigabyte data bundles and multiple deployment steps that have been automated to be installed and configured as new releases become available [26]. The resulting deployed image can be instantiated by StackStorm to process the incoming genomic data deposited on secure cloud storage locations on demand, without idle or wasted CPU cycles, enabling UMCCR to grow as patient numbers increase.

**Figure 4:**
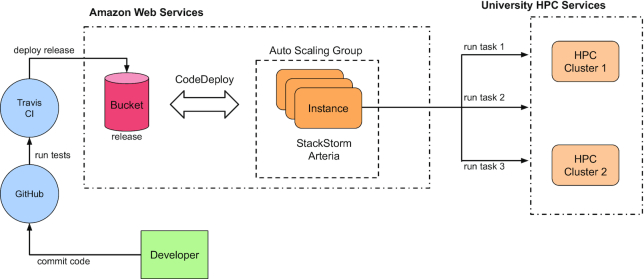
UMCCR Arteria cloud infrastructure. When a commit is pushed to our GitHub repository and validated by TravisCI, it proceeds to our autoscaling group “arteria,” which subsequently deploys cloud instances, incorporating the new Arteria and StackStorm code changes. After changes are deployed, any incoming events, such as a new sequencing run being completed, are handled by this newly deployed code and data are copied from the sequencers to our university HPC center for further downstream processing with bcbio [24].

The Arteria solution is deployed to Amazon Web Services, using a CI/CD (Continuous Integration and Continuous Deployment) approach. The entire system is developed and published to GitHub, where incoming changes are automatically tested using the continuous integration system TravisCI. If all tests are successful, new code is deployed into the cloud using the vendor's mechanisms, allowing new changes to be brought into production without human intervention. This is illustrated by Fig. 4.

Utilizing a hybrid approach takes advantage of the high reliability and flexibility provided by commercial IT providers, while still being able to carry out heavy and potentially sensitive computations in an in-house environment. In this environment Arteria offers a modular and reusable framework that eases common integration and middleware issues with systems like LIMS, data management, and archival.

## Discussion and Conclusion

In this article, we describe the automation system Arteria, which is built on top of the StackStorm automation platform and the Mistral workflow service. Arteria has successfully been adopted by 3 separate sequencing core facilities, where it forms a crucial part of their infrastructure, thus demonstrating the usefulness of the approach.

Arteria presents an approach to managing the full breadth of the operational aspects surrounding sequencing center operations. It manages *when* as well as *how* certain processes are to be carried out. Through the use of StackStorm as the orchestration engine, we have both a framework for the development of new functionality and a unified user interface for the system operators. The use of workflows at the process level, through Mistral, reduces the need for additional documentation and lowers the risk of human errors. Furthermore, the use of workflows allows for changes to the process to be code reviewed, in accordance with best practices in software development. Finally, the use of micro-services at the execution level has enabled a greater degree of flexibility in the execution model, a clear separation of responsibilities between services, as well as the integration of existing software. Being able to easily integrate existing software into the system has enabled quicker implementation because it lowers the burden of validation for, e.g., the ISO/IEC 17025 standard accreditation.

Arteria takes advantage of existing open source tools and aims at creating an avenue for collaboration between sequencing core facilities. We believe that decoupling process from execution, especially the micro-services developed within the Arteria project, could serve as fertile ground for collaboration. The stand-alone nature of the micro-services means that it should be possible for anyone interested to pick them up and include them in their own operations. Prior to beginning work on Arteria in 2015 we carried out an informal survey of possible systems to build upon. The main contender to StackStorm at the time appeared to be Airflow [13]. However, we judged that StackStorm had better documentation, which would make it a better choice of platform for us.

One important aspect when adopting novel software is whether the software can be expected to be maintained over time. While there is currently no explicit funding for the Arteria project, it is already being used across multiple sequencing core facilities. This means that there is already an existing community that can be approached for support. Furthermore, by building Arteria on larger and company-backed projects (i.e., Stackstorm and Mistral), the maintenance burden for most of the underlying functionality is deferred to those projects and should mitigate the risk of the Arteria project being abandoned. Finally, the separation of the systems into independent components means that different parts of the system, e.g., the micro-services, can still be used, even if support for other parts were to be discontinued. In fact, the different parts of the system, e.g., the workflow engine, are themselves interchangeable; thus, the entire project does not rest on the continued maintenance of a single component in it.

It should be noted that because the Arteria project does not provide out-of-the-box solutions, but rather demonstrates how facilities can build their own to suit their particular process, adopters of the Arteria system should expect to update their own systems as necessary. This includes StackStorm, workflows, and the micro-services being used.

We recognize that this type of approach has a higher initial overhead than, e.g., an orchestration system based on scripts and cron-tab entries. This overhead includes additional hardware requirements (current production hardware requirements are a quad core CPU, >16 GB RAM, 40G of storage) and increased system complexity. The increased complexity means that personnel resources, with experience in Linux systems and software development, must be dedicated to the development and maintenance of an Arteria system. This is particularly true in the initial implementation phase of a new system. We estimate that to implement Arteria at the SNP&SEQ Technology Platform we dedicated 2 full-time–equivalents (FTE) over a period of 1.5 years. This has since decreased, and we estimate that today we dedicate 0.5 FTEs to developing and maintaining the system. The exact time spent per month varies widely, from close to 0 in months when we only apply minor upstream updates to StackStorm to 1 FTE in months when we add new functionality.

Considering the costs of both hardware and personnel, a core facility considering implementing an Arteria system (or any similar system) needs to weigh the costs versus the benefits of adopting it. In our opinion, important things to consider include:

- the number of sequencing runs that need to be processed in a year
- the amount of manual work that can be accepted per sequencing run
- turnaround time requirements (e.g., time to response to a clinician in clinical sequencing applications)
- diversity of processes, e.g., supporting many different sequencing applications that require different processing workflows
- complexity of workflows, i.e., how many tasks make up a workflow, and whether the workflows contain branching, conditionals, etc.
- the need for traceability, i.e., how important it is to be able to log each action taken in the system for future audit

Considering the above, there are multiple scenarios in which it could be a good idea to adopt an Arteria system because it may help in dealing with these issues. For example, a sequencing facility having a large number of sequencing runs per year, e.g., 1 per day, and high demands on rapid turnaround time, may benefit from using this approach. Another example could be that a sequencing facility has few sequencing runs but many processes and complex workflows. Also in this scenario, an Arteria system might be a good option. Note that the amount of data is not a primary consideration in this decision. Many small sequencing runs tend to produce more work than few large ones.

There are situations where spending the required resources does not make sense. For example, if you need to process relatively few sequencing runs, e.g., 1 per week, and all those runs can be processed in a relatively straightforward manner, the additional overhead introduced by adopting an Arteria system may not be worth the investment.

However, under the right circumstances, we are confident that the additional overhead pays off, by proving a solid and extensible framework for developing new functionality in accordance with a core facility's needs, without requiring extensive changes to the existing infrastructure.

In the future we expect to keep improving on the Arteria system. In particular we aim at improving the micro-services. Two improvements we are planning on are (i) the standardization of the micro-services APIs and (ii) the implementation of https support in the micro-services, to ensure that communication to and from the services is encrypted. However, we still recommend running the micro-services behind a reverse proxy, in order to handle authentication/authorization, and encryption at a central point. Furthermore, we recommend that Arteria be run in a private network (physical or virtual), for increased security. A complete discussion on the securing of web applications is, however, out of scope for this article, and we recommend that any deployment of an Arteria system be secured according to industry standards.

In conclusion, the Arteria system presents a scalable and flexible solution to the operational issues of data management and analysis faced by sequencing core facilities. All components of Arteria are open source and available to the wider community [17], and the validity of the approach is demonstrated by the fact that multiple centers have Arteria systems handling their operations. Finally, we hope that the design described here can be instructive for anyone who needs to implement an orchestration system in the context of a sequencing core facility, or elsewhere.

## Availability of Supporting Source Code and Requirements

Project name: The Arteria project

Project home page: https://arteria-project.github.io/

Operating system(s): Linux

Programming language: Python

Other requirements: Docker, Docker Compose, make

License: MIT

SciCrunch RRID:SCR_017460

The package arteria-packs, which features Docker images for the system described in this article, is available for download at: https://github.com/arteria-project/arteria-packs.

## Availability of Supporting Data and Materials

The data set supporting the results of this article, “Reduced size Illumina NovaSeq runfolder,” is available in the Zenodo repository [23]. A snapshot of the code is also available via the *GigaScience* GigaDB repository [27].

## Additional files

Supplementary Figure 1. Example of information flow between levels in Arteria workflow.

giz135_GIGA-D-19-00180_Original_Submission

giz135_GIGA-D-19-00180_Revision_1

giz135_Response_to_Reviewer_Comments_Original_Submission

giz135_Reviewer_1_Report_Original_Submission Jorge Andrade, Ph.D. -- 6/20/2019 Reviewed

giz135_Reviewer_2_Report_Original_Submission Morgan Taschuk -- 7/29/2019 Reviewed

giz135_Supplemental_Figure

## Abbreviations

API: Application Programming Interface; bp: base pairs; CD: continuous delivery; CI: continuous integration; CPU: central processing unit; FTE: full-time equivalent; HPC: high-performance computing; LIMS: laboratory information management system; MPS: massively parallel sequencing; REST: Representational State Transfer; UMCCR: University of Melbourne Center for Cancer Research; WGS: whole-genome sequencing; WTS: whole-transcriptome sequencing.

## Competing Interests

The authors declare that they have no competing interests.

## Funding

This work was supported by the R&D group at the SNP&SEQ Technology Platform in Uppsala. This facility is part of the National Genomics Infrastructure (NGI) Sweden and Science for Life Laboratory. The SNP&SEQ Platform is also supported by the Swedish Research Council and the Knut and Alice Wallenberg Foundation. UMCCR work was funded through the Australian Genomics Health Alliance, which is supported by the National Health and Medical Research Council (GNT1113531). Clinical Genomics Uppsala is part of the Diagnostics Development platform within Science for Life Laboratory. Work at the Clinical Genomics Uppsala facility was also supported by grants from the Akademiska University Hospital (ALF-717721).

## Authors' contributions

J.D., J.H., S.S., and P.L. conceptualized the system. J.D., J.H., S.S., M.L., P.S., R.V.G., and P.L. contributed source code. All authors have contributed to writing the manuscript. All authors read and approved the final manuscript.
