# Supplementary material for: Arteria: An automation system for a sequencing core facility
Source: Gigascience. 2019 Dec 11;8(12):giz135. doi: 10.1093/gigascience/giz135 (PMC6905352; doi:10.1093/gigascience/giz135)

GigaScience

Arteria: An automation system for a sequencing core facility

--Manuscript Draft--

**Manuscript Number:**	GIGA-D-19-00180
**Full Title:**	Arteria: An automation system for a sequencing core facility
**Article Type:**	Technical Note
**Funding Information:**	Science for Life Laboratory — Not applicable<br>Swedish Research Council (N/A) — Not applicable<br>Knut and Alice Wallenberg Foundation (N/A) — Not applicable<br>National Health and Medical Research Council (GNT1113531) — Not applicable<br>Akademiska University Hospital (ALF-717721) — Not applicable

Abstract:

Background

In recent years, nucleotide sequencing has become increasingly instrumental in both research and clinical settings. This has led to an explosive growth in sequencing data produced worldwide. As the amount of data increases, so does the need for automated solutions for data processing and analysis. The concept of workflows has gained favour in the bioinformatics community, but there is little in the scientific literature describing end-to-end automation systems. Arteria is an automation system which aims at providing a solution to the data-related operational challenges which face sequencing core facilities.

Findings

Arteria is built on existing open-source technologies, with a modular design allowing for a community-driven effort to create plug-and-play micro-services. In this article we describe the system, elaborate on the underlying conceptual framework, and present an example implementation. Arteria can be reduced to three conceptual levels: orchestration (using an event-based model of automation), process (the steps involved in processing sequencing data, modelled as workflows), and execution (using a series of RESTful micro-services). This creates a system which is both flexible and scalable. Arteria-based systems have been successfully deployed at three sequencing core facilities. The Arteria Project code, written largely in Python, is available as open source software, and more information can be found at: https://arteria-project.github.io/

Conclusions

We describe the Arteria system and the underlying conceptual framework, demonstrating how this model can be used to automate data handling and analysis in the context of a sequencing core facility.

**Corresponding Author:**	Johan Dahlberg, Ph.D.<br>Department of Medical Sciences, Molecular Medicine and Science for Life Laboratory, Uppsala University, Uppsala, Sweden<br>SWEDEN
**Corresponding Author Secondary Information:**	
**Corresponding Author's Institution:**	Department of Medical Sciences, Molecular Medicine and Science for Life Laboratory, Uppsala University, Uppsala, Sweden
**Corresponding Author's Secondary Institution:**	
**First Author:**	Johan Dahlberg, Ph.D.

Powered by Editorial Manager® and ProduXion Manager® from Aries Systems Corporation

First Author Secondary Information:	
Order of Authors:	Johan Dahlberg, Ph.D.
	Johan Hermansson
	Steinar Sturlaugsson
	Mariya Lysenkova
	Patrik Smeds
	Claes Ladenvall, PhD
	Roman Valls Guimera
	Florian Reisinger
	Oliver Hofmann, PhD
	Pontus Larsson, PhD
Order of Authors Secondary Information:	
Additional Information:	
Question	Response
Are you submitting this manuscript to a special series or article collection?	No
**Experimental design and statistics**<br><br>Full details of the experimental design and statistical methods used should be given in the Methods section, as detailed in our Minimum Standards Reporting Checklist. Information essential to interpreting the data presented should be made available in the figure legends.<br><br>Have you included all the information requested in your manuscript?	No
If not, please give reasons for any omissions below.<br><br>as follow-up to "**Experimental design and statistics**<br><br>Full details of the experimental design and statistical methods used should be given in the Methods section, as detailed in our Minimum Standards Reporting Checklist. Information essential to interpreting the data presented should be made available in the figure legends.	The manuscript describes a software system rather than a traditional experiment.

Powered by Editorial Manager® and ProduXion Manager® from Aries Systems Corporation

Have you included all the information requested in your manuscript?<br><br>"	
**Resources**<br><br>A description of all resources used, including antibodies, cell lines, animals and software tools, with enough information to allow them to be uniquely identified, should be included in the Methods section. Authors are strongly encouraged to cite Research Resource Identifiers (RRIDs) for antibodies, model organisms and tools, where possible.<br><br>Have you included the information requested as detailed in our Minimum Standards Reporting Checklist?	Yes
**Availability of data and materials**<br><br>All datasets and code on which the conclusions of the paper rely must be either included in your submission or deposited in publicly available repositories (where available and ethically appropriate), referencing such data using a unique identifier in the references and in the "Availability of Data and Materials" section of your manuscript.<br><br>Have you have met the above requirement as detailed in our Minimum Standards Reporting Checklist?	Yes

Manuscript Click here to download Manuscript Arteria manuscript.docx

Arteria: An automation system for a sequencing core facility

Johan Dahlberg[1*], Johan Hermansson[1], Steinar Sturlaugsson[1], Mariya Lysenkova[1], Patrik Smeds[2], Claes Ladenvall[2], Roman Valls Guimera[3], Florian Reisinger[3], Oliver Hofmann[3], Pontus Larsson[1]

[1]Department of Medical Sciences, Molecular Medicine and Science for Life Laboratory, Uppsala University, Uppsala, Sweden

[2]Department of Immunology, Genetics and Pathology and Science for Life Laboratory, Uppsala University, Uppsala, Sweden

[3]University of Melbourne Center for Cancer Research, University of Melbourne, Melbourne, Australia

[*] Corresponding author (johan.dahlberg@medsci.uu.se)

Abstract

Background

In recent years, nucleotide sequencing has become increasingly instrumental in both research and clinical settings. This has led to an explosive growth in sequencing data produced worldwide. As the amount of data increases, so does the need for automated solutions for data processing and analysis. The concept of workflows has gained favour in the bioinformatics community, but there is little in the scientific literature describing end-to-end automation systems. Arteria is an automation system which aims at providing a solution to the data-related operational challenges which face sequencing core facilities.

Findings

Arteria is built on existing open-source technologies, with a modular design allowing for a community-driven effort to create plug-and-play micro-services. In this article we describe the system, elaborate on the underlying conceptual framework, and present an example implementation. Arteria can be reduced to three conceptual levels: *orchestration* (using an event-based model of automation), *process* (the steps involved in processing sequencing data, modelled as workflows), and *execution* (using a series of RESTful micro-services). This creates a system which is both flexible and scalable. Arteria-based systems have been successfully deployed at three sequencing core facilities. The Arteria Project code, written largely in Python, is available as open source software, and more information can be found at: https://arteria-project.github.io/

Conclusions

We describe the Arteria system and the underlying conceptual framework, demonstrating how this model can be used to automate data handling and analysis in the context of a sequencing core facility.

Keywords: automation, sequencing, orchestration, workflows

Findings

Challenges in, and approaches to, processing sequencing data

Nucleotide sequencing is the practice of determining the order of bases of the nucleic acid sequences that form the foundation of all known forms of life. It has been hugely successful as a research tool, used to understand basic biology [1–3], and is also applied as a tool for precision medicine [4]. Major technological advances during the last decade have enabled high throughput approaches for massively parallel sequencing (MPS) [5]. The amount of data generated globally from MPS has boomed in recent

years, and has been projected to reach a yearly production of 10^{21} base-pairs per year by 2025, demanding 2-40 Exa-bytes (10^{18}) per year of storage [6]. This massive expansion places new demands on how data is analyzed, stored and distributed.

Much of this nucleotide sequencing is carried out at sequencing core facilities, which perform sequencing as a service. The kinds of services provided vary widely, but most facilities provide at least some processing of raw sequencing data, typically conversion to a standard fastq format at a minimum [7]. More advanced bioinformatic analysis may involve passing the data through a pipeline of software processes. Such processes often require manual initiation or intervention, creating a significant overhead of labor and increased turnaround times.

Automation of both laboratory and computational procedures is crucial in order for a sequencing facility to scale the number of samples processed. Furthermore, automated processes reduce the risk of human errors, which contributes to higher quality data.

However, there are challenges to automating these processes. Despite the high standardization of lab protocols, a number of factors create a combinatorial situation that makes every lab unique, including small variation in procedures, infrastructure and surrounding systems. This requires the development of bespoke solutions to manage specific situations. One example of these custom solutions are laboratory information management systems (LIMS), which are used to track the laboratory procedures that a sample is subjected to, and to perform surrounding utility tasks such as generating instruction files for pipetting robots. LIMS are often based on extensible platforms in which specific laboratory protocols can be implemented.

The potential complexity in the laboratory will often extend into the computational environment. The specific nature of the infrastructure and services offered places wide-ranging demands on the

computational systems developed to support management and high-throughput analysis of sequencing data. This has led most sequencing core facilities to develop their own custom solutions to this problem, and these are often highly coupled to the infrastructure and process of that particular core facility [8].

These systems need to be designed not only to support the analysis of data, but to address additional aspects associated with operating a sequencing facility. Examples include automatically starting data processing when a sequencing run has finished, archiving of data to remote storage, and selective data removal. These *operational* aspects have not been thoroughly investigated in the scientific literature, but are essential when taking a bird's-eye view of the complete process of refining raw MPS data to scientific results on a high-throughput scale. Tackling these issues involves examination of how higher level orchestration, integration, and management of workflows can be done in an efficient yet flexible manner, while providing a clear enough understanding of the system so that changes can be implemented with minimal mental overhead and risk of breaking existing functionality. Arteria fills a niche by providing a systematic way of approaching the operational aspects of data management and analysis of MPS data.

In recent years there has been an increased interest in workflow systems, both in academia [8–11] and in industry [12, 13]. Typically these systems model a workflow as a directed acyclic graph of dependencies between computational tasks. The core concepts that define workflows, as used here, are defined in table 1.

Table 1: Definitions

**Term**	**Description**
Action	A computational unit of work, e.g. processing a file or inserting data into a database. This is sometimes referred to as a task.

Process	A set of steps that have to be finished to achieve a particular goal, e.g. delivering data to a user. A process can include automated and manual steps.
Workflow	A workflow models a process, as a number of *actions* following each-other. This can be described by a directed acyclic graph.

These workflows are often designed to be executed on a per-project or per-sample level, with parameters being provided manually by the operator. This model is well-suited for processing large amounts of data, where all samples in a project can be analyzed the same way. However, for institutions that provide sequencing as a service to multiple users or projects, this type of system does not scale well, due to the need for manual intervention at different stages of the process.

One example of a system addressing the operational challenges outlined above in the context of a sequencing core facility is described by Cuccuru et. al [14]. They describe a system with a central automator that handles orchestration of the processes in an event-based manner, utilizing the Galaxy platform [15] as a separate workflow manager. The Galaxy platform provides a web-based interface, making bioinformatic analysis accessible to users who lack the training to use command-line tools. The system's automator is based on daemons monitoring a RabbitMQ [16] based event-queue. While this system shares ideas with the Arteria system, it does not have the same focus on decoupling the system from the implementation at the facility in question. Furthermore, the Arteria system benefits from building on top of existing industry standards for event-based automation systems rather than building these from scratch.

Herein, we describe the automation system Arteria, which is available as open-source software at: https://github.com/arteria-project. Arteria utilizes the open source automation platform StackStorm [17] for event-based orchestration, the Mistral [18] workflow engine for process modelling, and Python micro-

services for action execution. Arteria has been successfully implemented at three separate sequencing core facilities to date: the SNP&SEQ Technology Platform at Science For Life Laboratory, the Clinical Genomics Uppsala at Science For Life Laboratory, and the University of Melbourne Center for Cancer Research.

System overview

The Arteria system is built with two existing open source technologies at its core: the StackStorm automation platform [17] and the Mistral workflow service [18]. By adopting existing open-source solutions and extending them for our domain, we are able to leverage the power of a larger open-source community. This has allowed us to focus on our specific use-case: automation of sequencing data processing.

The Arteria system can be divided into three conceptual levels, a model that has been adopted from StackStorm: the orchestration level, the process level and the execution level (figure 1).

At the highest level, the orchestration level, StackStorm serves as the central point of automation. It provides both command-line and web interfaces through which an operator can interact with the system. It utilizes an event-based model to decide when actions should be triggered. For example, the completion of a sequencing run is an event that may trigger further actions to be taken by the system.

At the process level, internal processes are modelled as workflows using the Mistral service. For example, a workflow triggered by the completion of a sequencing run may then carry out basic processing, gather quality control data, and transfer the data to a high-performance computing resource.

Finally, at the execution level, actions are carried out. This level includes multiple modes of execution, ranging from a shell command on a local or remote machine, to interaction with surrounding systems such as a LIMS, to invoking a micro-service. The final mode, the micro-service, is the one favoured by Arteria. The advantages of the micro-service approach include system flexibility and the decoupling of implementation details of the execution from the process which invokes it.

This separation of the system into levels makes the Arteria system easier to deconstruct, and places implementation details at the correct level of abstraction. In addition, Arteria enforces a separation of concerns that makes it easier to update or replace individual components, without having to make large changes to the system as a whole. This creates a flexible system which is able to meet the scaling demands placed on sequencing core facilities, where protocols are modified and new instrumentation is routinely implemented.

Event-based orchestration

At the orchestration level, Arteria uses StackStorm to coordinate tasks. A core concept of StackStorm is its event-based model of automation (see figure 2). It utilizes sensors to detect events from the environment. A typical example is a sequencing instrument finishing a run. The event parameters are then passed through a rule layer that decides which, if any, action should be taken. This simple yet powerful abstraction makes the Arteria system and its behaviour simple to deconstruct. In addition to triggering actions in response to sensor events, an operator can manually initiate an action via a command line or web interface.

Furthermore, StackStorm provides per-action monitoring capabilities. Each action taken by the Arteria system is assigned a unique id, allowing operators to follow the progress of processes in the system. An additional advantage is the ability to create audit trails, which are both useful internally and required to

accredit systems, e.g. it is required by the European quality standard ISO/IEC 17025 [19] under which the SNP&SEQ Technology Platform operates. Finally, providing a centralized interface to the underlying processes means that operators require less knowledge of the underlying components and direct access to fewer systems, which is an advantage from a security perspective.

Modelling processes as workflows

At the process level, the process of a particular use case is modeled using the Mistral workflow language. Mistral uses a declarative yaml syntax to define a workflow, which allows for the definition of complicated dependency structures. It supports the use of conditionals, forking (defining multiple tasks that must be run after the completion of a given task) and joining (the synchronization of multiple parallel workflow branches and aggregation of their data). It will execute actions that do not have dependencies on each other concurrently. This simple and powerful syntax has the additional advantage of serving as a human-readable documentation of the modelled process. Modelling a process as a workflow can mean formalizing the documentation of an existing process into a Mistral workflow, thus reducing the amount of manual work required as well as reducing the risk of human errors.

Micro-services provide a flexible execution model

Finally, at the execution level, any action that needs to be carried out by the system is performed. Arteria favors the use of single-purpose micro-service executors, and these provide the actual functionality and logic for performing the actions. These micro-services are invoked from the process level through an HTTP API, making the communication simple and allowing for easy integration with other services. An example of such a micro-service is the one provided by Arteria to run the preprocessing program Illumina bcl2fastq [20], which processes the raw data produced by an Illumina sequencing instrument and converts it to the industry standard fastq-format. However, a micro-service is not required; the Arteria approach is flexible enough that it supports running a shell command or invoking another service, e.g. a LIMS.

Using micro-services as the primary execution mode increases the flexibility of the Arteria system as the implementation details of *how* something is run is decoupled from *when* it is run. Furthermore, such micro-services can be reused across systems, or even centers, creating an avenue for reuse and collaboration, which sets the Arteria approach apart from other sequencing core facility systems that are typically tightly coupled to the process and infrastructure of the sequencing core facility that developed it.

Finally, decoupling the execution layer has allowed us to build simple interfaces for existing software, thus significantly reducing the burden of having to reimplement components that have been used reliably for a long time in operation.

Implementation

Arteria is comprised of publicly-available software packages, written largely in Python, that can be grouped into two components. The first component is arteria-packs, a plugin for the orchestration engine Stackstorm, which acts as a starting template for individual core facilities to build their own implementation.

The second component is a series of single-responsibility REST micro-services, comprised of both general-interest packages that can be reused across sequencing core facilities, and tailor-made services that cater to a single facility's specific needs. These provide specific functionality, such as running the Illumina bcl2fastq program, checking if a runfolder is ready to be analyzed, or removing data once certain criteria are met. These microservices, and others, are available from https://github.com/arteria-project.

The package arteria-packs is available for download at: https://github.com/arteria-project/arteria-packs (the accompanying README provides detailed installation instructions). The purpose of this package is

two-fold: first, to act as a demo illustrating a minimal but complete Arteria system: second, to serve as a template for sequencing core facilities to build upon in developing their own Arteria implementations.

During the setup process for arteria-packs, Docker is used to create an environment that is comprised of Stackstorm, its dependencies, and three general-interest Arteria microservices: arteria-runfolder, arteria-bcl2fastq and checkQC.

The repository provides the sample units detailed in table 2.

Table 2. Descriptions of concepts in arteria-packs sample implementation.

**Concept**	**Definition**	**arteria-packs implementation**
Actions	encapsulate system tasks	Micro-services arteria-runfolder, arteria-bcl2fastq and checkQC
Workflows	tie actions together	Mistral workflow defined in workflow_bcl2fastq_and_checkqc.yaml
Sensors	pick up events from the environment	RunfolderSensor defined in runfolder_sensor.yaml, which detects runfolders ready for processing.
Rules	parse events from sensors and determine if an action or a workflow should be initiated	Defined in when_runfolder_is_ready_start_bcl2fastq.yaml; fires bcl2fastq

		workflow when a runfolder is ready

The workflow, defined in workflows/bclfastq_and_checkqc.yaml, outlines the following actions to detect and process a runfolder:

- get_runfolder_name
- mark_as_started
- start_bcl2fastq
- poll_bcl2fastq
- checkqc
- mark_as_done
- mark_as_error

In arteria-packs, the example workflow operates as follows. The runfolder_sensor routinely polls the arteria-runfolder service to retrieve information about unprocessed runfolders. When the service returns the runfolder_ready event, the Stackstorm sensor rule when_runfolder_is_ready_start_bcl2fastq is triggered, initiating arteria-bcl2fastq, which demultiplexes data and converts the binary base call (BCL) format to FASTQ. The Stackstorm instance will poll arteria-bcl2fastq until it receives a “done” status. The arteria-runfolder service is then invoked to mark the runfolder with the state of “done” or “error”.

To test the workflow, a runfolder containing Illumina-generated sequencing data may be placed in the docker-mountpoints/monitored-folder directory. A sample runfolder is available at: https://doi.org/10.5281/zenodo.1204292.

A command can then be run to initiate the workflow manually. Alternatively, the rule when_runfolder_is_ready_start_bcl2fastq can be enabled, allowing automatic processing of any ready runfolder. Refer to the repository README for details.

The arteria-packs repository serves as a starting point for a sequencing core facility to implement its own actions, workflows, sensors and rules.

Deployment scenario and usage statistics

SNP&SEQ Technology Platform

The SNP&SEQ Technology Platform sequencing core facility at Science for Life Laboratory provides sequencing and genotyping as a service to the Swedish research community. Projects from a wide variety of fields are accepted, ranging from clinical research projects to environmental sciences. In addition, the facility provides a large number of assays; some examples are DNA, RNA and bisulfite-converted library preparations and sequencing, as well as the sequencing of libraries prepared by users.

At the SNP&SEQ Technology Platform, the Arteria system is deployed in a distributed environment (see figure 3) and orchestrates actions across a local cluster of 10 nodes used for storage and preliminary analysis with 208 cores and 120 TB of storage capacity, as well as a high-performance computing cluster at the Uppsala Multidisciplinary Center for Advanced Computational Science (UPPMAX) high-performance computing center with 4000 cores and 2.1 PB storage. This system is fully capable of supporting the fleet of 8 Illumina sequencers (2 NovaSeq, 3 HiSeqX, 1 HiSeq 2500, 1 MiSeq, and 1 iSeq) which are currently in use at the SNP&SEQ Technology Platform.

The SNP&SEQ Technology platform uses Arteria workflows that automatically pick up data as the sequencing instrument finishes a sequencing run, convert it into the industry standard FASTQ format, check it against a set of quality criteria, upload data to off-site archiving, and transfer it to the high-performance computing cluster. Other workflows include data delivery to users on a per-run or per-project basis, synchronizing data between local and remote systems, weekly report generation and automatic read-back tests of archived sequencing data.

Adopting Arteria at the SNP&SEQ Technology platform has reduced the amount of manual work required to process sequencing data. It has also provided a convenient interface through which most of our internal processes can now be monitored. Furthermore, the detailed process and error logs provided by Arteria are used to build reporting dashboards, allowing staff to see which processes are bottlenecks and to set concrete, quantitative automation goals.

Since being deployed at the SNP&SEQ Technology Platform, the Arteria system has been used to process more than 50000 samples and 715 projects, which corresponds to ~1213 Tera-bases of sequencing data.

Clinical Genomics, Uppsala

At the Clinical Genomics Uppsala (Science for Life Laboratory) MPS analyses are developed and implemented for clinical use with the aim to improve diagnostics and allow for targeted treatments. The turnaround time for certain samples must be short, preferably one to two hours from when the facility obtains raw data to when processed data is sent to clinical staff for manual interpretation and reporting.

Raw sequence data is produced at the Uppsala University Hospital, but at present the hospital lacks the capacity to analyse MPS data within the required time. To overcome this, the bioinformatic processing is performed on a private cluster with 8 nodes at Uppsala University. Because of this setup our analyses

include transfers between networks, including the more secure hospital network that only allows for communication to be initiated from within the hospital.

Our Arteria implementation is deployed in a docker environment, and consists of Arteria and in-house developed workflows. It is routinely used to push data from the hospital to our cluster located in the Uppsala University Network. When raw data is transferred to the cluster, Arteria subsequently initiates analyses on the cluster, pulls back results into the hospital network and archives data. We also use Arteria to send out emails when results are available or when an error occurs in the sample processing.

The need for a minimal turnaround time and our network limitations makes Arteria and its micro-services a key component for us. The solution automates several tasks that previously were done manually, reducing labour-intensive and time consuming repetitive steps in our pipelines. After being deployed, the system has processed 2176 samples originating from 362 sequence runs. Apart from automating a repetitive task, we estimate that we now save ~2 hours of bioinformatician working time for each sequencing run and slightly improved our turnaround time.

We are currently working on implementing the system on remaining pipelines that we have set up. In the near future we will also implement a solution for the automatic conversion of raw sequence data from sequencing machines to the standard FASTQ format, using the arteria-bcl2fastq micro-service.

The University of Melbourne Center for Cancer Research (UMCCR)

The University of Melbourne Center for Cancer Research (UMCCR) aims to improve cancer patient outcome and enable personalized medicine through rapid whole genome (WGS) and transcriptome (WTS) sequencing of tumor samples. Operating in a clinical, accredited environment requires reproducible and traceable data management which the Center implements through Arteria, providing

crucial automation, error notifications, provenance, LIMS control, centralized monitoring and orchestration.

Rapid WGS/WTS data is generated by Illumina's NovaSeq platform and processed through the bcbio framework [21] both at commercial cloud provider (Amazon Web Service) and traditional high-performance computing (HPC) centers within Australia. Arteria automates primary data movement between computational environments and handles distribution of results to a cBio portal, long term archival storage facilities and other downstream services such as automatic report generation through the Personal Cancer Genome Reporter [22]. This software suite includes multi-gigabyte data bundles and multiple deployment steps which have been automated to be installed and configured as new releases become available [23]. The resulting deployed image can be instantiated by StackStorm to process the incoming genomic data deposited on secure cloud storage locations on demand, without idle or wasted CPU cycles, enabling UMCCR to grow as patient numbers increase.

The Arteria solution is deployed to Amazon Web Services, using a CI/CD (Continuous Integration and Continuous Deployment) approach. The entire system is developed and published to GitHub, where incoming changes are automatically tested using the continuous integration system TravisCI. If all tests are successful, new code is deployed into the cloud using the vendor's mechanisms, allowing new changes are brought into production without human intervention. This is illustrated by figure 4.

Utilizing a hybrid approach takes advantage of the high reliability and flexibility provided by commercial IT providers, while still being able to carry out heavy and potentially sensitive computations in an in-house environment. In this environment Arteria offers a modular and reusable framework that eases common integration and middleware issues with systems like LIMS, data management and archival.

Discussion and conclusion

In this paper, we describe the automation system Arteria, which is built on top of the StackStorm automation platform and the Mistral workflow service. Arteria has successfully been adopted by three separate sequencing core facilities, where it forms a crucial part of their infrastructure, thus demonstrating the usefulness of the approach.

Arteria presents an approach to managing the full breadth of the operational aspects surrounding sequencing center operations. It manages *when* as well as *how* certain processes are to be carried out. Through the use of StackStorm as the orchestration engine, we are able to both have a framework for the development of new functionality as well as providing a unified user interface to the system operators.The use of workflows at the process level, through Mistral, reduces the need for additional documentation and lowers the risk of human errors. Furthermore, the use of workflows allows for changes to the process to be code reviewed, in accordance with best practices in software development. Finally, the use of micro-services at the execution level has enabled a greater degree of flexibility in the execution model, a clear separation of responsibilities between services, as well as the integration of existing software. Being able to easily integrate existing software into the system has enabled quicker implementation as it lowers the burden of validation for e.g. the ISO/IEC 17025 standard accreditation.

Arteria takes advantage of existing open source tools and aims at creating an avenue for collaboration between sequencing core facilities. We believe that decoupling process from execution, especially the micro-services developed within the Arteria project, could serve as fertile ground for collaboration. The stand-alone nature of the micro-services means that it should be possible for anyone interested to pick them up and include them in their own operations.

We recognize that this type of approach has a higher initial overhead than, for example, an orchestration system based on scripts and cron-tab entries. This overhead includes additional hardware requirements (current production hardware requirements are: a quad core CPU, >16GB RAM, 40G of storage) and increased system complexity. This increase in system complexity can make debugging more difficult. However, in the long run we are confident that the additional overhead pays off, by proving a solid and extensible framework for developing new functionality in accordance with our core facility's needs, without requiring extensive changes to the existing infrastructure.

In conclusion, the Arteria system presents a scalable and flexible solution to the operational issues of data management and analysis faced by sequencing core facilities. All components of Arteria are open source and available to the wider community (https://github.com/arteria-project), and the validity of the approach is demonstrated by the fact that multiple centers have Arteria systems handling their operations. Finally, we hope that the design described here can be instructive for anyone who needs to implement an orchestration system in the context of a sequencing core facility, or elsewhere.

Availability of supporting source code and requirements

Project name: The Arteria project

Project home page: https://arteria-project.github.io/

Operating system(s): Linux

Programming language: Python

Other requirements: Docker, Docker Compose, make

License: MIT

The package arteria-packs, which features Docker images for the system described in this paper, is available for download at: https://github.com/arteria-project/arteria-packs

Availability of Supporting Data

The data set supporting the results of this article, “Reduced size Illumina NovaSeq runfolder”, is available in the Zenodo repository, with the unique persistent identifier 1204292: https://doi.org/10.5281/zenodo.1204292.

Declarations

List of abbreviation

MPS	Massively parallel sequencing
LIMS	Laboratory information management systems
CI	Continuous integration
CD	Continuous delivery

Ethics approval and consent to participate

Not applicable

Consent for publication

Not applicable

Availability of data and material

Not applicable.

Competing interests

The authors declare that they have no competing interests.

Funding

This work was supported by the R&D group at the SNP&SEQ Technology Platform in Uppsala. This facility is part of the National Genomics Infrastructure (NGI) Sweden and Science for Life Laboratory. The SNP&SEQ Platform is also supported by the Swedish Research Council and the Knut and Alice Wallenberg Foundation. UMCCR work was funded through the Australian Genomics Health Alliance which is supported by the National Health and Medical Research Council (GNT1113531). The Clinical Genomics Uppsala is part of the Diagnostics Development platform within Science for Life Laboratory. Work at the Clinical Genomics Uppsala facility was also supported by grants from the Akademiska University Hospital (ALF-717721).

Authors' contributions

JD, JH, SS, and PL conceptualized the system. JD, JH, SS, ML, PS, RVG, and PL contributed source code. All authors have contributed to writing the manuscript. All authors read and approved the final manuscript.

Acknowledgements

The entire bioinformatics team at the SNP&SEQ Technology Platform has contributed to the Arteria project with feedback during its adoption. Especially Monika Brandt, Matilda Åslin and Sara Ekberg have provided extremely useful feedback based on their daily operations of the system, as well as contributing code to the project. Parts of the computations for the Arteria Project were performed on resources

provided by SNIC through Uppsala Multidisciplinary Center for Advanced Computational Science (UPPMAX).

We would like to acknowledge Samuel Lampa and Jessica Nordlund for reading this text and providing highly valuable comments, which have been instrumental in developing this manuscript.

Figures

Figure 1 - An overview of the conceptual levels of the Arteria project.

Figure 2 - Description of the StackStorm event model. Sensors will perceive events in the environments, e.g. a file being created or a certain time of day it occurs. This passes information to the rule layer where the data is evaluated and depending on which, if any, criteria are fulfilled one or more actions are triggered. Actions can be single commands or full workflows to be executed.

Figure 3 - Schematic view of a system deployment scenario, showing how data is written to the local storage and compute nodes from the sequencing machines, and how the system uses information and resources from multiple sources to coordinate the process. The operator can then monitor and control the processes from the single interface provided at the master automation node.

Figure 4 - UMCCR arteria cloud infrastructure. When a commit is pushed to our github repository and validated by TravisCI, it proceeds to our autoscaling group "arteria" which subsequently deploys cloud instances, incorporating the new Arteria and StackStorm code changes. After changes are deployed, any incoming event such as a new sequencing run being completed, are handled by this newly deployed code and data is copied from the sequencers to our university HPC center for further downstream processing with bcbio [21].

References

1. Lander ES, Linton LM, Birren B, Nusbaum C, Zody MC, Baldwin J, et al. Initial sequencing and analysis of the human genome. Nature. 2001;409:860–921.

2. Spang A, Saw JH, Jørgensen SL, Zaremba-Niedzwiedzka K, Martijn J, Lind AE, et al. Complex archaea that bridge the gap between prokaryotes and eukaryotes. Nature. 2015;521:173–9.

3. 1000 Genomes Project Consortium, Auton A, Brooks LD, Durbin RM, Garrison EP, Kang HM, et al. A global reference for human genetic variation. Nature. 2015;526:68–74.

4. Ashley EA. Towards precision medicine. Nat Rev Genet. 2016;17:507–22.

5. Goodwin S, McPherson JD, McCombie WR. Coming of age: ten years of next-generation sequencing technologies. Nat Rev Genet. 2016;17:333–51.

6. Stephens ZD, Lee SY, Faghri F, Campbell RH, Zhai C, Efron MJ, et al. Big Data: Astronomical or Genomical? PLoS Biol. 2015;13:e1002195.

7. Spjuth O, Bongcam-Rudloff E, Dahlberg J, Dahlö M, Kallio A, Pireddu L, et al. Recommendations on e-infrastructures for next-generation sequencing. Gigascience. 2016;5:26.

8. Spjuth O, Bongcam-Rudloff E, Hernández GC, Forer L, Giovacchini M, Guimera RV, et al. Experiences with workflows for automating data-intensive bioinformatics. Biol Direct. 2015;10:43.

9. Lampa S, Alvarsson J, Spjuth O. Towards agile large-scale predictive modelling in drug discovery with flow-based programming design principles. J Cheminform. 2016;8:67.

10. Leipzig J. A review of bioinformatic pipeline frameworks. Brief Bioinform. 2017;18:530–6.

11. Amstutz P, Crusoe MR, Tijanić N, Chapman B, Chilton J, Heuer M, et al. Common Workflow

Language, v1.0. Figshare. 2016. doi:10.6084/m9.figshare.3115156.v2.

12. Spotify. Luigi. GitHub. 2017. https://github.com/spotify/luigi. Accessed 24 Jan 2017.

13. Apache. https://github.com/apache/incubator-airflow. GitHub. 2017. https://github.com/apache/incubator-airflow. Accessed 24 Jan 2017.

14. Cuccuru G, Leo S, Lianas L, Muggiri M, Pinna A, Pireddu L, et al. An automated infrastructure to support high-throughput bioinformatics. In: 2014 International Conference on High Performance Computing & Simulation (HPCS). IEEE; 2014. p. 600–7.

15. Afgan E, Baker D, van den Beek M, Blankenberg D, Bouvier D, Čech M, et al. The Galaxy platform for accessible, reproducible and collaborative biomedical analyses: 2016 update. Nucleic Acids Res. 2016;44:W3–10.

16. RabbitMQ - Messaging that just works. http://www.rabbitmq.com/. Accessed 7 Feb 2018.

17. StackStorm. StackStorm/st2. GitHub. 2017. https://github.com/StackStorm/st2. Accessed 24 Jan 2017.

18. Mistral. 2016. https://wiki.openstack.org/wiki/Mistral. Accessed 16 Aug 2016.

19. ISO/IEC 17025:2005 - General requirements for the competence of testing and calibration laboratories. 2014. https://www.iso.org/standard/39883.html. Accessed 10 Apr 2017.

20. Illumina. bcl2fastq2 Conversion Software v2.17. http://support.illumina.com/downloads/bcl2fastq-conversion-software-v217.html. Accessed 15 Aug 2016.

21. Chapman B. bcbio-nextgen. https://github.com/chapmanb/bcbio-nextgen. Accessed 8 Feb 2018.

22. Nakken S, Fournous G, Vodák D, Aasheim LB, Myklebost O, Hovig E. Personal Cancer Genome Reporter: variant interpretation report for precision oncology. Bioinformatics. 2017.

doi:10.1093/bioinformatics/btx817.

23. Personal Cancer Genome Reporter deployment recipes. https://github.com/umccr/pcgr-deploy. Accessed 8 Feb 2018.

figure 1

Click here to download Figure figure1.pdf

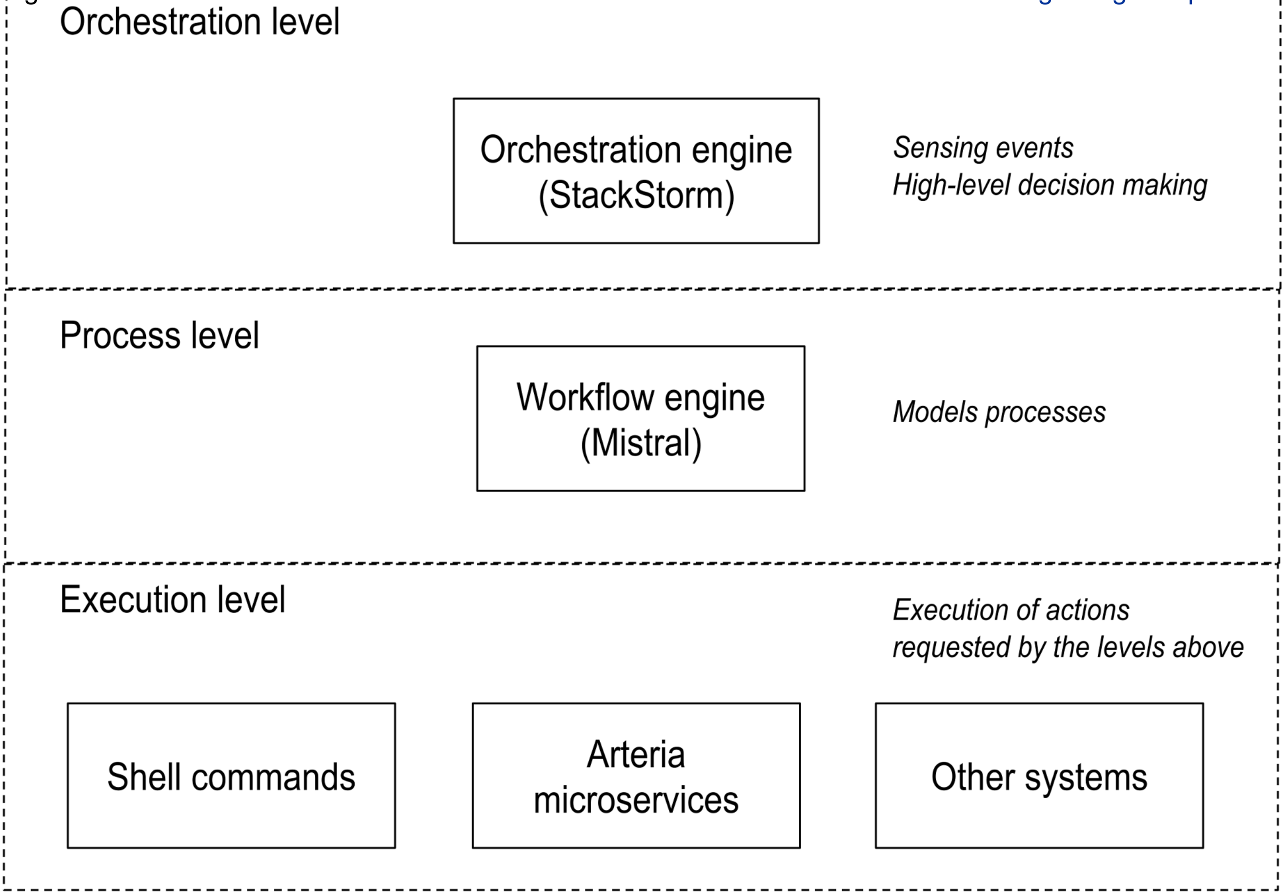

figure 2

Click here to download Figure figure2.pdf

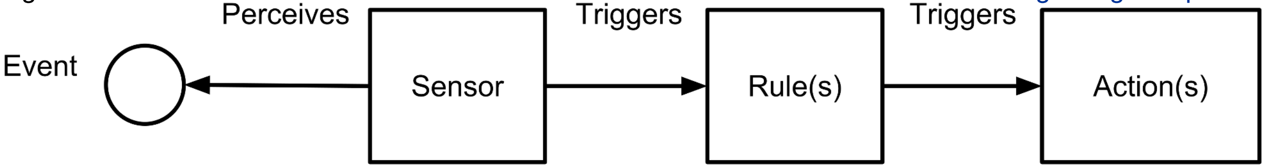

figure 3

Click here to download Figure figure3.pdf

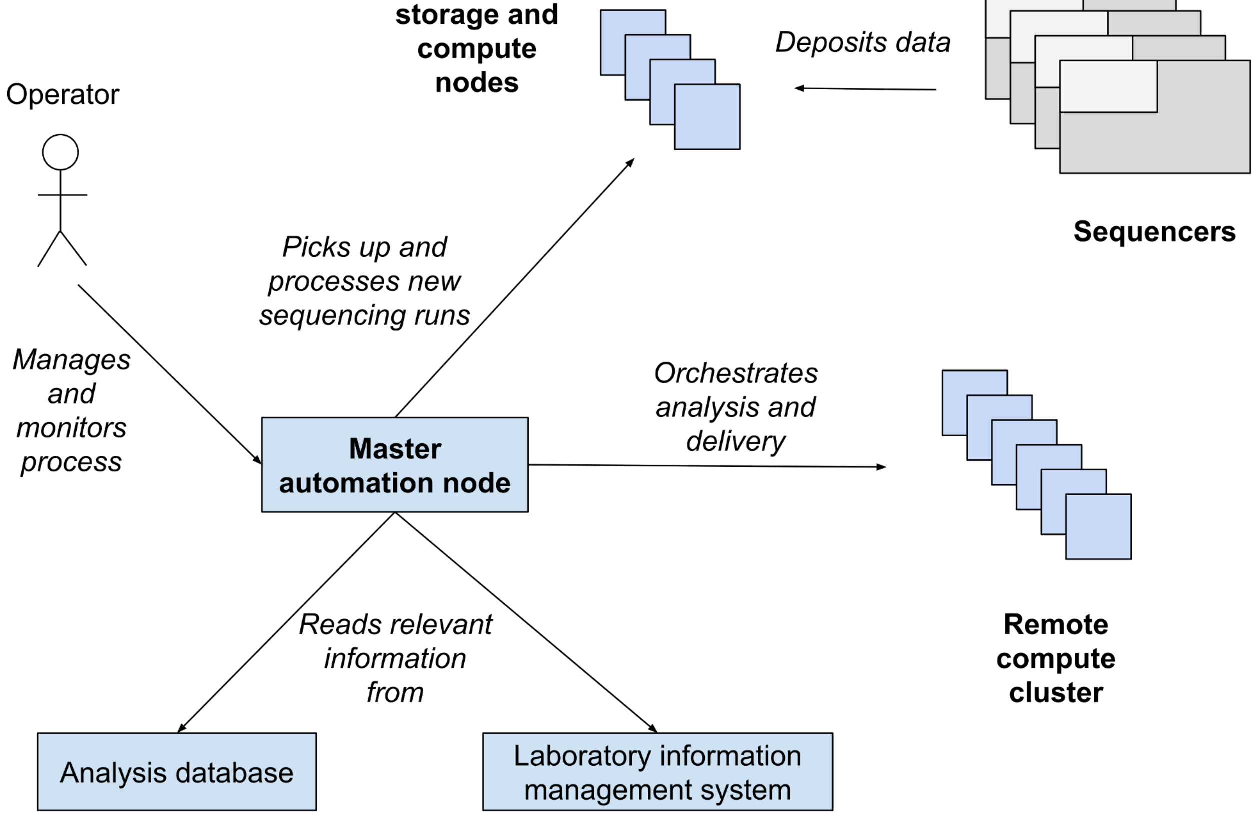

figure 4

Click here to download Figure figure4.pdf

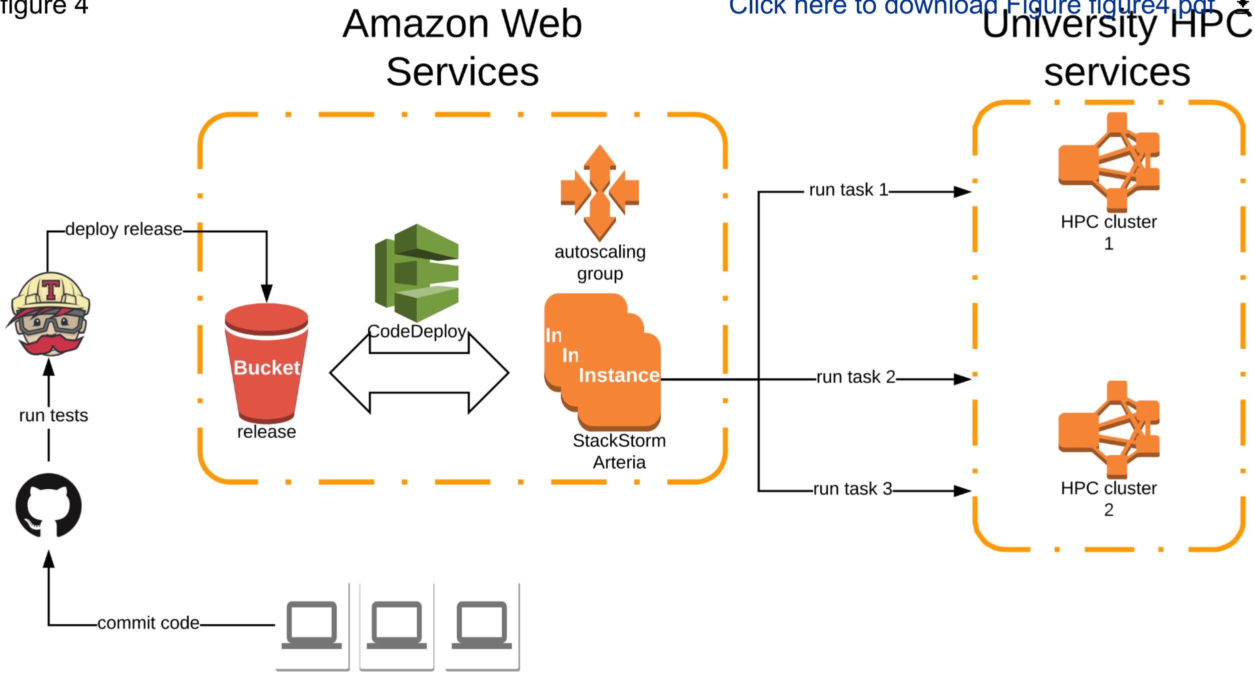

Supplement: giz135_GIGA-D-19-00180_Original_Submission [file giz135_giga-d-19-00180_original_submission.pdf]
