# Supplementary material for: Arteria: An automation system for a sequencing core facility
Source: Gigascience. 2019 Dec 11;8(12):giz135. doi: 10.1093/gigascience/giz135 (PMC6905352; doi:10.1093/gigascience/giz135)
Supplement: giz135_GIGA-D-19-00180_Revision_1 [file giz135_giga-d-19-00180_revision_1.pdf]

GigaScience

Arteria: An automation system for a sequencing core facility

--Manuscript Draft--

**Manuscript Number:**	GIGA-D-19-00180R1
**Full Title:**	Arteria: An automation system for a sequencing core facility
**Article Type:**	Technical Note
**Funding Information:**	Science for Life Laboratory — Not applicable<br>Swedish Research Council (N/A) — Not applicable<br>Knut and Alice Wallenberg Foundation (N/A) — Not applicable<br>National Health and Medical Research Council (GNT1113531) — Not applicable<br>Akademiska University Hospital (ALF-717721) — Not applicable

Abstract:

Background

In recent years, nucleotide sequencing has become increasingly instrumental in both research and clinical settings. This has led to an explosive growth in sequencing data produced worldwide. As the amount of data increases, so does the need for automated solutions for data processing and analysis. The concept of workflows has gained favour in the bioinformatics community, but there is little in the scientific literature describing end-to-end automation systems. Arteria is an automation system which aims at providing a solution to the data-related operational challenges which face sequencing core facilities.

Findings

Arteria is built on existing open-source technologies, with a modular design allowing for a community-driven effort to create plug-and-play micro-services. In this article we describe the system, elaborate on the underlying conceptual framework, and present an example implementation. Arteria can be reduced to three conceptual levels: orchestration (using an event-based model of automation), process (the steps involved in processing sequencing data, modelled as workflows), and execution (using a series of RESTful micro-services). This creates a system which is both flexible and scalable. Arteria-based systems have been successfully deployed at three sequencing core facilities. The Arteria Project code, written largely in Python, is available as open source software, and more information can be found at: https://arteria-project.github.io/

Conclusions

We describe the Arteria system and the underlying conceptual framework, demonstrating how this model can be used to automate data handling and analysis in the context of a sequencing core facility.

**Corresponding Author:**	Johan Dahlberg, Ph.D.<br>Department of Medical Sciences, Molecular Medicine and Science for Life Laboratory, Uppsala University, Uppsala, Sweden<br>SWEDEN
**Corresponding Author Secondary Information:**	
**Corresponding Author's Institution:**	Department of Medical Sciences, Molecular Medicine and Science for Life Laboratory, Uppsala University, Uppsala, Sweden

Powered by Editorial Manager® and ProduXion Manager® from Aries Systems Corporation

Corresponding Author's Secondary Institution:	
First Author:	Johan Dahlberg, Ph.D.
First Author Secondary Information:	
Order of Authors:	Johan Dahlberg, Ph.D.
	Johan Hermansson
	Steinar Sturlaugsson
	Mariya Lysenkova
	Patrik Smeds
	Claes Ladenvall, PhD
	Roman Valls Guimera
	Florian Reisinger
	Oliver Hofmann, PhD
	Pontus Larsson, PhD
Order of Authors Secondary Information:	
Response to Reviewers:	Dear editor and reviews,<br><br>Thank you for your insightful comments. They have really helped in improving the manuscript. We have made changes to the manuscript in accordance with your suggestions. In order to make it easier to follow how we have addressed your suggestions, we have written answers after each paragraph, surrounded by “>>>”.<br><br>Once again, many thanks for the excellent feedback, and we hope that you feel that the changes we have made are to your satisfaction.<br><br>Yours sincerely,<br>Johan Dahlberg, PhD<br><br>Reviewer #1<br>The authors present a computational framework built over existing open-source technologies like the StackStorm, to develop an event-driven automation platform for processing sequencing data. Automation and workflow management still represents a significant challenge on many Sequencing facilities, there here presented system is a step in the right direction and should capture the attention of the community.<br><br>Other workflow management systems such as snakemake and nextflow are available, the community how these systems compare with the here presented framework? for instance,<br>the system presented here uses the Python ecosystem. The Python ecosystem is a mess to work with when it comes to 3rd party libraries that need to be installed on HPC. The installation often requires environment management, not all of which are solvable with virtualenv's or conda. Nextflow installs seamlessly on any system that has Java 8.<br><br>>>><br>We do not explicitly compare Arteria, and in particular Mistral, to other workflow system, in the article because the problem we are trying to solve is a slightly different one. Snakemake and Nextflow are focused on running bioinformatical tools. Typically command line tools which have files are their inputs and outputs. Consequently, they are well suited to solving that type of problem. Arteria focuses instead of solving issues of operational automation, e.g. moving data between compute clusters, and updating databases. A step in that process may be to use e.g. Nextflow or Snakemake to carry out more complex analysis workflows. So, as part of an Arteria workflow, one might trigger a Nextflow workflow to be run, and then fetch the files and upload them to a file server for long term storage.

We have tried to clarify this distinction in the “Challenges in, and approaches to, processing sequencing data” section now.

When it comes to the Python ecosystem, we acknowledge the points that you bring up. However, we believe that any programing language comes with its own set of pros and cons. In this case we have decided to build upon Python mainly for two reasons. Firstly, Python is the language used by the native StackStorm APIs, so deviating from the would be inconvenient. Secondly, Python is a language with a high adoption rate in the bioinformatics community, which means that it should be easier for others to understand and modify our code.
>>>

Besides what a tool can or cannot do, potential users need to check the quality of the documentation, whether it is actively developed and maintained, how many developers contribute to it, and size of the user base. The authors clearly describe two case-studies at the SNP&SEQ Technology Platform sequencing core facility at Science for Life Laboratory and the Clinical Genomics, Uppsala, however, it will be important to know what is the plan for continued funding, development, and maintenance for this system, this is very important in order to make it an attractive and sustainable alternative for the community.

>>>
This is a good point. Arteria, like many projects of this type, lack dedicated funding. At the SNP&SEQ Technology Platform we have deeply invested in it, and base our entire operations on it. However, we recognize that this is not the same as having a guaranteed that the project will be maintained indefinitely.

We hope that by basing Arteria on an existing framework which is backed by a much larger community, some of the risk of the project becoming orphaned is mitigated. As long as StackStorm is maintained, the orchestration and workflow levels of the systems should get updates, while it would be incumbent on users of the system to provide updates to the microservices they use.

We have added a paragraph in the discussion that deals with this point now.
>>>

A case study is presented to demonstrate the system's usability. Illumina bcl2fastq tools is used to perform the demultiplexing (dividing sequence reads into separate files for each index tag/sample) and generating the fastq data files required for downstream analysis, for some Illumina sequencing platforms, this step is carried out automatically using the onboard PC. For others, this step is just a simple Linux command line. In order to really demonstrate the workflow management abilities of this platform, the authors should incorporate other downstream analysis steps like raw data quality control with FASTQC, mapping, feature quantification (for RNA-Seq) or Variant Calling (for DNA-seq) in their demo/example case study.

>>>
As discussed above, the purpose of Arteria is focused on operational rather than analytical problems. We hope that the explanation and accompanying changes described above have adequately addressed your concerns on this topic. We have added a picture to the supplementary materials which exemplifies the type of workflows that Arteria is focused on.
>>>

How the system handles the necessary user-defined parameters for a particular task? for instance, the bcl2fastq process usually needs a sample sheet - a simple comma separated file (csv) with the library chemistry, sample names and the index tag used for each sample, in addition to some other metrics describing the run, this will, of course, needs to be customized for different users, per-run or per project. In a similar manner, the incorporation of further downstream analysis steps on the pipeline will require a user-defined sample description table (i.e. for DEG detection).

>>>

We have added a clarification to the “Event-based orchestration” section, to make it explicit that when starting an action (e.g. a workflow) manually, the user may (and sometimes must) provide parameters.
>>>

How the system handles conditional creation of events based on the input data? for instance, Snakemake allows for conditional creation of the DAG and conditional execution of different code based on the input. Is this feature supported by the system?

>>>
Yes, this feature is supported by the system. We describe it in the section “Modelling processes as workflows”:

“It supports the use of conditionals, forking (defining multiple tasks that must be run after the completion of a given task) and joining (the synchronization of multiple parallel workflow branches and aggregation of their data).”
>>>

Does the system feature singularity support with the singularity directive? This is an important feature since not all potential HPC users will have root access to deploy Docker containers in their infrastructures.

>>>
No. The system does not have direct support for singularity, and is aimed at users, which at least in part control their own infrastructure, and can install software, open ports in firewalls, etc. However, that said, services can be run through singularity using the service concept (https://sylabs.io/guides/3.0/user-guide/running_services.html). We view singularity, like docker, or other container technologies as complementary to the micro-services, not as something that can replace them.
>>>

Reviewer #2
The authors describe the Arteria system for sequencing core automation. Arteria is a mechanism for fully automating the analysis parts of a sequencing core, including fastq generation and QC, data transfer/archiving, and data removal, and more generally for thinking about operational aspects of a sequencing core in a structured, site-agnostic way. Arteria is based on other open source technologies: StackStorm for orchestration and Mistral workflow language. The authors argue that by depending on these external packages, they are free to concentrate on the sequencing specific requirements. As a result, Arteria is not a self-contained piece of software. Instead it is all of the 'glue code' required to use StackStorm and Mistral for the purposes of a sequencing centre, as well as the specific microservices that are REST interfaces for launching processes like bcl2fastq.

This is important work that has been little-spoken-of in the bioinformatics analysis community and I think that Arteria contributes greatly to the discussion and ongoing improvement. The concepts, separation of concerns, and focus on good, secure design are fundamental to the way we think about sequencing core automation. Relying on open source software is a good idea to reduce the amount of overhead and reliance on individual sequencing centres. The flexibility of the system to adapt to new centres is exemplified by the three separate use-cases. I was quite impressed by the ability to run a CU/CD approach for one of the use-cases.

It would be interesting and contribute to overall understanding of the system to have a figure or text description of a specific process from end to end, e.g. what kicks off when the 'sequencing is done' sensor is triggered. There is a very short description, but it would be interesting to see the system process diagram of when StackStorm and Mistral are contacted with what information, when the services launched contact other services, where reports/emails are generated, etc. All of this is essentially already in the Github project, in a less friendly way. It would be especially interesting if you included details such as what happens when something goes wrong, for example, if the LIMS has the incorrect molecular index and bcl2fastq fails.

>>>

Powered by Editorial Manager® and ProduXion Manager® from Aries Systems Corporation

We have added a diagram showing how information flows between different levels in the system now. This is however, rather large, so we have opted to add it as a supplementary figure rather than to add it into the manuscript proper.
>>>

The discussion section feels thin, with little to no comparison of their method to other methods. Some of what I expected here has been included in the 'Findings' section, but the discussion should be an opportunity to honestly examine what has been done in context with other methods. The authors do mention that there isn't much published on this topic, but a short examination of the benefits of this method compared to other workflow engines, other event schedulers, other bioinformatics methods would be appreciated. A few suggestions are included below.

>>>
Thank you for providing excellent feedback on the discussion. We will address the different suggestions below, to make it easier to follow up on.
>>>

Who is this system for? Should centres need 2 Novaseqs before they consider Arteria? What is the minimum size of operation that would make this infeasible? On the other end, how much can these systems really scale?

>>>
We have added a section to the discussion, that elaborates on what we think are important things to consider when deciding whether to adopt an Arteria system or not. Naturally, it is difficult to provide a hard boundary for when we would adopt recommendation since there are so many factors to consider. However, we hope that you find the points of discussion useful.
>>>

Automation systems are rarely published because every core requires small variation in procedure, infrastructure, surrounding systems. Each use case mentions how much time is saved, but how much effort is it for an institution to set up these systems? An estimate with number of people and months/years of effort would be sufficient.

>>>
We have included estimates of how much time we have dedicated to the development and maintenance of the system at the SNP&SEQ Technology platform, both in the initial, implementation phase, as well as now that we are mainly maintaining the system.
>>>

The authors do not provide any justification for why they are using StackStorm and Mistral in particular. What benefits do these services offer compared to other orchestration and workflow engines? What are the main competitors?

>>>
Prior to starting work on Arteria in 2015 we did an informal survey of systems that we thought could fulfill the needs that we saw. One feature in particular that made StackStorm stand out was the use of sensors to detect events in the environment - while there are many workflow systems, from or brief survey, few seemed to support this way of automatically starting workflows. The main competitor at the time was Airflow, but at that time, at least the StackStorm documentation was superior to the Airflow documentation, and that made us select StackStorm. We have added a paragraph on this in the discussion now.
>>>

One thing I am always concerned about when it comes to using other software packages is whether it will be supported long term, and what happens if or when support is removed for it. How entangled are Arteria's systems with StackStorm and Mistral? What happens when one or both of them are updated?

>>>
Concerning long term support, Arteria, like many projects of this type, lack dedicated

funding. At the SNP&SEQ Technology Platform we have deeply invested in it, and base our entire operations on it. However, we recognize that this is not the same as having a guaranteed that the project will be maintained indefinitely.

We hope that by basing Arteria on an existing framework which is backed by a much larger community, some of the risk of the project becoming orphaned is mitigated. As long as StackStorm is maintained, the orchestration and workflow levels of the systems should get updates, while it would be incumbent on users of the system to provide updates to the microservices they use.

The separation between levels in the Arteria model, should further mitigate the risk. For example, one can still use the micro-services without using Mistral as a workflow engine, and one could switch out the workflow engine used, without making changes to the micro-service, etc.

We have added a paragraph in the discussion that deals with this point now.
>>>

What future work is expected on the system? Is there any maintenance expected, for example when there is new version of Mistral, or is each sequencing site on their own?

>>>
We expect to maintain the example implementation (github.com/arteria-project/arteria-packs), as well as the micro-services associated with the project. However, since most sequencing facilities will implement their own custom workflows, each site will have to update StackStorm, Mistral, etc according to their own needs. We have added a paragraph to the discussion to clarify that.
>>>

About the Arteria microservices. There are several implemented microservices based on Tornado, a python package for implementing web services. These seem to have a bespoke shape, i.e. each microservice is different from the next. With everything else so defined, I found this an interesting oversight. I would like a short discussion of what kind of information a microservice needs provided. Have the authors considered any of the interfaces from the Global Alliance for Global Health (GA4GH)? In particular, the workflow execution schema or task execution schema? Was there any consideration of using Docker or other container technology instead of microservices?

>>>
We completely agree that standardization of the APIs of the micro-services is an important area where we could improve in the future. We have looked at the specifications provided by the GA4GH, however, we are not convinced that any of the Task Execution Service API or the Workflow Execution Service API, are a perfect match. The former focuses on abstracting the submission of tasks to e.g. a cluster scheduler, and the latter on running workflows based on e.g. Common Workflow Language. We can see a scenario in which the service in turn communicates with a service implementing either API, but the services themselves are meant to abstract away many of the details that are required from those APIs.

We consider container technologies to be complementary to the use of micro-service. For example, in the example implementation of an Arteria, we run the micro-services in docker containers which are orchestrated by docker compose.

We have added a section to the discussion, about this.
>>>

The execution-level microservices are implemented on HTTP (unencrypted) microservices. Especially since several of the use-cases involve not only the analysis, but also the transfer of clinical human data, there should be a small note about securing these systems against unauthorized access and interception. What architecture is necessary in order to keep these secure? What should a new site absolutely not do?

>>>

Powered by Editorial Manager® and ProduXion Manager® from Aries Systems Corporation

	Adding https support to the micro-services is something that is on our roadmap. However, we recommend using a reverse-proxy to handle encryption, authentication/authorization. In our internal setup we use Kong for this purpose. In general we do not recommend running Arteria in open networks, but rather recommend that it is run in a section of private network (physical or virtual). We have added a section to the discussion on this topic, however, we do feel that a complete discussion on the security of web-applications is out of scope for this paper.<br>>>><br><br>All in all, this paper is a great contribution to this discussion. As the authors say, not much has been published in this domain before and that's an enormous oversight. Hopefully this paper can begin the discussion around such automation systems. I absolutely support publication after addressing a few of the points above.
Additional Information:	
Question	Response
Are you submitting this manuscript to a special series or article collection?	No
**Experimental design and statistics**<br><br>Full details of the experimental design and statistical methods used should be given in the Methods section, as detailed in our Minimum Standards Reporting Checklist. Information essential to interpreting the data presented should be made available in the figure legends.<br><br>Have you included all the information requested in your manuscript?	No
If not, please give reasons for any omissions below.<br><br>as follow-up to "**Experimental design and statistics**<br><br>Full details of the experimental design and statistical methods used should be given in the Methods section, as detailed in our Minimum Standards Reporting Checklist. Information essential to interpreting the data presented should be made available in the figure legends.<br><br>Have you included all the information requested in your manuscript?<br><br>"	The manuscript describes a software system rather than a traditional experiment.

Powered by Editorial Manager® and ProduXion Manager® from Aries Systems Corporation

**Resources**<br><br>A description of all resources used, including antibodies, cell lines, animals and software tools, with enough information to allow them to be uniquely identified, should be included in the Methods section. Authors are strongly encouraged to cite Research Resource Identifiers (RRIDs) for antibodies, model organisms and tools, where possible.<br><br>Have you included the information requested as detailed in our Minimum Standards Reporting Checklist?	Yes
**Availability of data and materials**<br><br>All datasets and code on which the conclusions of the paper rely must be either included in your submission or deposited in publicly available repositories (where available and ethically appropriate), referencing such data using a unique identifier in the references and in the “Availability of Data and Materials” section of your manuscript.<br><br>Have you have met the above requirement as detailed in our Minimum Standards Reporting Checklist?	Yes

Manuscript

Click here to access/download;Manuscript;Arteria manuscript GigaScience - post-review submission.docx

Click here to view linked References

Arteria: An automation system for a sequencing core facility

Johan Dahlberg[1*], Johan Hermansson[1], Steinar Sturlaugsson[1], Mariya Lysenkova[1], Patrik Smeds[2], Claes Ladenvall[2], Roman Valls Guimera[3], Florian Reisinger[3], Oliver Hofmann[3], Pontus Larsson[1]

[1]Department of Medical Sciences, Molecular Medicine and Science for Life Laboratory, Uppsala University, Uppsala, Sweden

[2]Department of Immunology, Genetics and Pathology and Science for Life Laboratory, Uppsala University, Uppsala, Sweden

[3]University of Melbourne Center for Cancer Research, University of Melbourne, Melbourne, Australia

[*] Corresponding author (johan.dahlberg@medsci.uu.se)

ORCID IDs:

Johan Dahlberg: 0000-0001-6962-1460; Claes Ladenvall: 0000-0002-7501-6598; Roman Valls Guimera: 0000-0002-0034-9697; Oliver Hofmann: 0000-0002-7738-1513; Pontus Larsson: 0000-0002-8597-5565

Abstract

Background

In recent years, nucleotide sequencing has become increasingly instrumental in both research and clinical settings. This has led to an explosive growth in sequencing data produced worldwide. As the amount of data increases, so does the need for automated solutions for data processing and analysis. The concept of workflows has gained favour in the bioinformatics community, but there is little in the scientific literature describing end-to-end automation systems. Arteria is an automation system which aims at providing a solution to the data-related operational challenges which face sequencing core facilities.

Findings

Arteria is built on existing open-source technologies, with a modular design allowing for a community-driven effort to create plug-and-play micro-services. In this article we describe the system, elaborate on the underlying conceptual framework, and present an example implementation. Arteria can be reduced to three conceptual levels: *orchestration* (using an event-based model of automation), *process* (the steps involved in processing sequencing data, modelled as workflows), and *execution* (using a series of RESTful micro-services). This creates a system which is both flexible and scalable. Arteria-based systems have been successfully deployed at three sequencing core facilities. The Arteria Project code, written largely in Python, is available as open source software, and more information can be found at: https://arteria-project.github.io/

Conclusions

We describe the Arteria system and the underlying conceptual framework, demonstrating how this model can be used to automate data handling and analysis in the context of a sequencing core facility.

Keywords: automation, sequencing, orchestration, workflows

Findings

Challenges in, and approaches to, processing sequencing data

Nucleotide sequencing is the practice of determining the order of bases of the nucleic acid sequences that form the foundation of all known forms of life. It has been hugely successful as a research tool, used to understand basic biology [1–3], and is also applied as a tool for precision medicine [4]. Major technological advances during the last decade have enabled high throughput approaches for massively parallel sequencing (MPS) [5]. The amount of data generated globally from MPS has boomed in recent years, and has been projected to reach a yearly production of 10^{21} base-pairs per year by 2025, demanding 2-40 Exa-bytes (10^{18}) per year of storage [6]. This massive expansion places new demands on how data is analyzed, stored and distributed.

Much of this nucleotide sequencing is carried out at sequencing core facilities, which perform sequencing as a service. The kinds of services provided vary widely, but most facilities provide at least some processing of raw sequencing data, typically conversion to a standard fastq format at a minimum [7]. More advanced bioinformatic analysis may involve passing the data through a pipeline of software processes. Such processes often require manual initiation or intervention, creating a significant overhead of labor and increased turnaround times.

Automation of both laboratory and computational procedures is crucial in order for a sequencing facility to scale the number of samples processed. Furthermore, automated processes reduce the risk of human errors, which contributes to higher quality data.

However, there are challenges to automating these processes. Despite the high standardization of lab protocols, a number of factors create a combinatorial situation that makes every lab unique, including small variation in procedures, infrastructure and surrounding systems. This requires the development

of bespoke solutions to manage specific situations. One example of these custom solutions are laboratory information management systems (LIMS), which are used to track the laboratory procedures that a sample is subjected to, and to perform surrounding utility tasks such as generating instruction files for pipetting robots. LIMS are often based on extensible platforms in which specific laboratory protocols can be implemented.

The potential complexity in the laboratory will often extend into the computational environment. The specific nature of the infrastructure and services offered places wide-ranging demands on the computational systems developed to support management and high-throughput analysis of sequencing data. This has led most sequencing core facilities to develop their own custom solutions to this problem, and these are often highly coupled to the infrastructure and process of that particular core facility [8].

These systems need to be designed not only to support the analysis of data, but to address additional aspects associated with operating a sequencing facility. Examples include automatically starting data processing when a sequencing run has finished, archiving of data to remote storage, and selective data removal. These *operational* aspects have not been thoroughly investigated in the scientific literature, but are essential when taking a bird's-eye view of the complete process of refining raw MPS data to scientific results on a high-throughput scale. Tackling these issues involves examination of how higher level orchestration, integration, and management of workflows can be done in an efficient yet flexible manner, while providing a clear enough understanding of the system so that changes can be implemented with minimal mental overhead and risk of breaking existing functionality. Arteria fills a niche by providing a systematic way of approaching the operational aspects of data management and analysis of MPS data.

In recent years there has been an increased interest in workflow systems, both in academia [8–11] and in industry [12, 13]. Typically, these systems model a workflow as a directed acyclic graph of

dependencies between computational tasks. The core concepts that define workflows, as used here, are defined in table 1.

Table 1: Definitions

**Term**	**Description**
Action	A computational unit of work, e.g. processing a file or inserting data into a database. This is sometimes referred to as a task.
Process	A set of steps that have to be finished to achieve a particular goal, e.g. delivering data to a user. A process can include automated and manual steps.
Workflow	A workflow models a process, as a number of *actions* following each-other. This can be described by a directed acyclic graph.

These workflows are often designed to be executed on a per-project or per-sample level, with parameters being provided manually by the operator. Furthermore, they typically focus tying together command line programs that have files as inputs and outputs. This model is well-suited for processing large amounts of data, where all samples in a project can be analyzed the same way. However, for institutions that provide sequencing as a service to multiple users or projects, this type of system does not scale well, due to the need for manual intervention at different stages of the process. Additionally, many sequencing core facilities will have workflows where file input/output is not the most natural solution. For example, updating a database or emailing reports will not generate files by default. Thus there is a need for systems to address these challenges, which can be thought of as operational rather than analytical.

One example of a system addressing the operational challenges outlined above in the context of a sequencing core facility is described by Cuccuru et. al [14]. They describe a system with a central automator that handles orchestration of the processes in an event-based manner, utilizing the Galaxy

platform [15] as a separate workflow manager. The Galaxy platform provides a web-based interface, making bioinformatic analysis accessible to users who lack the training to use command-line tools. The system's automator is based on daemons monitoring a RabbitMQ [16] based event-queue. While this system shares ideas with the Arteria system, it does not have the same focus on decoupling the system from the implementation at the facility in question. Furthermore, the Arteria system benefits from building on top of existing industry standards for event-based automation systems rather than building these from scratch.

Herein, we describe the automation system Arteria, which is available as open-source software at: https://github.com/arteria-project. Arteria utilizes the open source automation platform StackStorm [17] for event-based orchestration, the Mistral [18] workflow engine for process modelling, and Python micro-services for action execution. Arteria has been successfully implemented at three separate sequencing core facilities to date: the SNP&SEQ Technology Platform at Science For Life Laboratory, the Clinical Genomics Uppsala at Science For Life Laboratory, and the University of Melbourne Center for Cancer Research.

System overview

The Arteria system is built with two existing open source technologies at its core: the StackStorm automation platform [17] and the Mistral workflow service [18]. By adopting existing open-source solutions and extending them for our domain, we are able to leverage the power of a larger open-source community. This has allowed us to focus on our specific use-case: automation of sequencing data processing.

The Arteria system can be divided into three conceptual levels, a model that has been adopted from StackStorm: the orchestration level, the process level and the execution level (figure 1). For an overview of how information flows between levels, see supplementary figure 1.

At the highest level, the orchestration level, StackStorm serves as the central point of automation. It provides both command-line and web interfaces through which an operator can interact with the system. It utilizes an event-based model to decide when actions should be triggered. For example, the completion of a sequencing run is an event that may trigger further actions to be taken by the system.

At the process level, internal processes are modelled as workflows using the Mistral service. For example, a workflow triggered by the completion of a sequencing run may then carry out basic processing, gather quality control data, and transfer the data to a high-performance computing resource.

Finally, at the execution level, actions are carried out. This level includes multiple modes of execution, ranging from a shell command on a local or remote machine, to interaction with surrounding systems such as a LIMS, to invoking a micro-service. The final mode, the micro-service, is the one favoured by Arteria. The advantages of the micro-service approach include system flexibility and the decoupling of implementation details of the execution from the process which invokes it.

This separation of the system into levels makes the Arteria system easier to deconstruct, and places implementation details at the correct level of abstraction. In addition, Arteria enforces a separation of concerns that makes it easier to update or replace individual components, without having to make large changes to the system as a whole. This creates a flexible system which is able to meet the scaling demands placed on sequencing core facilities, where protocols are modified and new instrumentation is routinely implemented.

Event-based orchestration

At the orchestration level, Arteria uses StackStorm to coordinate tasks. A core concept of StackStorm is its event-based model of automation (see figure 2). It utilizes sensors to detect events from the environment. A typical example is a sequencing instrument finishing a run. The event parameters are then passed through a rule layer that decides which, if any, action or workflow should be started. If an action or workflow should be started, sufficient parameters need to be passed on by the rule layer. This simple yet powerful abstraction makes the Arteria system and its behaviour simple to understand. In addition to triggering actions in response to sensor events, an operator can manually initiate an action via a command line or web interface. When manually initiating an action, the operator must provide the parameters necessary to start the action, as well as any other optional parameters to modify the action's default behaviour.

Furthermore, StackStorm provides per-action monitoring capabilities. Each action taken by the Arteria system is assigned a unique id, allowing operators to follow the progress of processes in the system. An additional advantage is the ability to create audit trails, which are both useful internally and required to accredit systems, e.g. it is required by the European quality standard ISO/IEC 17025 [19] under which the SNP&SEQ Technology Platform operates. Finally, providing a centralized interface to the underlying processes means that operators require less knowledge of the underlying components and direct access to fewer systems, which is an advantage from a security perspective.

Modelling processes as workflows

At the process level, the process of a particular use case is modeled using the Mistral workflow language. Mistral uses a declarative yaml syntax to define a workflow, which allows for the definition of complicated dependency structures. It supports the use of conditionals, forking (defining multiple tasks that must be run after the completion of a given task) and joining (the synchronization of multiple parallel workflow branches and aggregation of their data). It will execute actions that do not have dependencies on each other concurrently. This simple and powerful syntax has the additional

advantage of serving as a human-readable documentation of the modelled process. Modelling a process as a workflow can mean formalizing the documentation of an existing process into a Mistral workflow, thus reducing the amount of manual work required as well as reducing the risk of human errors.

Micro-services provide a flexible execution model

Finally, at the execution level, any action that needs to be carried out by the system is performed. Arteria favors the use of single-purpose micro-service executors, and these provide the actual functionality and logic for performing the actions. These micro-services are invoked from the process level through an HTTP API, making the communication simple and allowing for easy integration with other services. An example of such a micro-service is the one provided by Arteria to run the preprocessing program Illumina bcl2fastq [20], which processes the raw data produced by an Illumina sequencing instrument and converts it to the industry standard FASTQ-format. However, a micro-service is not required; the Arteria approach is flexible enough that it supports running a shell command or invoking another service, e.g. a LIMS.

Using micro-services as the primary execution mode increases the flexibility of the Arteria system as the implementation details of *how* something is run is decoupled from *when* it is run. Furthermore, such micro-services can be reused across systems, or even centers, creating an avenue for reuse and collaboration, which sets the Arteria approach apart from other sequencing core facility systems that are typically tightly coupled to the process and infrastructure of the sequencing core facility that developed it.

Finally, decoupling the execution layer has allowed us to build simple interfaces for existing software, thus significantly reducing the burden of having to re-implement components that have been used reliably for a long time in operation.

Implementation

Arteria is comprised of publicly-available software packages, written largely in Python, that can be grouped into two components. The first component is arteria-packs, a plugin for the orchestration engine Stackstorm, which acts as a starting template for individual core facilities to build their own implementation.

The second component is a series of single-responsibility REST micro-services, comprised of both general-interest packages that can be reused across sequencing core facilities, and tailor-made services that cater to a single facility's specific needs. These provide specific functionality, such as running the Illumina bcl2fastq program, checking if a runfolder is ready to be analyzed, or removing data once certain criteria are met. These microservices, and others, are available from https://github.com/arteria-project.

The package arteria-packs is available for download at: https://github.com/arteria-project/arteria-packs (the accompanying README provides detailed installation instructions). The purpose of this package is two-fold: first, to act as a demo illustrating a minimal but complete Arteria system: second, to serve as a template for sequencing core facilities to build upon in developing their own Arteria implementations.

During the setup process for arteria-packs, Docker is used to create an environment that is comprised of Stackstorm, its dependencies, and three general-interest Arteria microservices: arteria-runfolder, arteria-bcl2fastq and checkQC.

The repository provides the sample units detailed in table 2.

Table 2. Descriptions of concepts in arteria-packs sample implementation.

**Concept**	**Definition**	**arteria-packs implementation**
Actions	encapsulate system tasks	Micro-services arteria-runfolder, arteria-bcl2fastq and checkQC
Workflows	tie actions together	Mistral workflow defined in workflow_bcl2fastq_and_checkqc.yaml
Sensors	pick up events from the environment	RunfolderSensor defined in runfolder_sensor.yaml, which detects runfolders ready for processing.
Rules	parse events from sensors and determine if an action or a workflow should be initiated	Defined in when_runfolder_is_ready_start_bcl2fastq.yaml; fires bcl2fastq workflow when a runfolder is ready

The workflow, defined in workflows/bclfastq_and_checkqc.yaml, outlines the following actions to detect and process a runfolder:

- get_runfolder_name
- mark_as_started
- start_bcl2fastq
- poll_bcl2fastq
- checkqc
- mark_as_done
- mark_as_error

In arteria-packs, the example workflow operates as follows. The runfolder_sensor routinely polls the arteria-runfolder service to retrieve information about unprocessed runfolders. When the service returns the runfolder_ready event, the Stackstorm sensor rule when_runfolder_is_ready_start_bcl2fastq is triggered, initiating arteria-bcl2fastq, which demultiplexes data and converts the binary base call (BCL) format to FASTQ. The Stackstorm instance will poll arteria-bcl2fastq until it receives a “done” status. The arteria-runfolder service is then invoked to mark the runfolder with the state of “done” or “error”.

To test the workflow, a runfolder containing Illumina-generated sequencing data may be placed in the docker-mountpoints/monitored-folder directory. A sample runfolder is available at: https://doi.org/10.5281/zenodo.1204292.

A command can then be run to initiate the workflow manually. Alternatively, the rule when_runfolder_is_ready_start_bcl2fastq can be enabled, allowing automatic processing of any ready runfolder. Refer to the repository README for details.

The arteria-packs repository serves as a starting point for a sequencing core facility to implement its own actions, workflows, sensors and rules.

Deployment scenario and usage statistics

SNP&SEQ Technology Platform

The SNP&SEQ Technology Platform sequencing core facility at Science for Life Laboratory provides sequencing and genotyping as a service to the Swedish research community. Projects from a wide variety of fields are accepted, ranging from clinical research projects to environmental sciences. In addition, the facility provides a large number of assays; some examples are DNA, RNA and bisulfite-

converted library preparations and sequencing, as well as the sequencing of libraries prepared by users.

At the SNP&SEQ Technology Platform, the Arteria system is deployed in a distributed environment (see figure 3) and orchestrates actions across a local cluster of 10 nodes used for storage and preliminary analysis with 208 cores and 120 TB of storage capacity, as well as a high-performance computing cluster at the Uppsala Multidisciplinary Center for Advanced Computational Science (UPPMAX) high-performance computing center with 4000 cores and 2.1 PB storage. This system is fully capable of supporting the fleet of 8 Illumina sequencers (2 NovaSeq, 3 HiSeqX, 1 HiSeq 2500, 1 MiSeq, and 1 iSeq) which are currently in use at the SNP&SEQ Technology Platform.

The SNP&SEQ Technology platform uses Arteria workflows that automatically pick up data as the sequencing instrument finishes a sequencing run, convert it into the industry standard FASTQ format, check it against a set of quality criteria, upload data to off-site archiving, and transfer it to the high-performance computing cluster. Other workflows include data delivery to users on a per-run or per-project basis, synchronizing data between local and remote systems, weekly report generation and automatic read-back tests of archived sequencing data.

Adopting Arteria at the SNP&SEQ Technology platform has reduced the amount of manual work required to process sequencing data. It has also provided a convenient interface through which most of our internal processes can now be monitored. Furthermore, the detailed process and error logs provided by Arteria are used to build reporting dashboards, allowing staff to see which processes are bottlenecks and to set concrete, quantitative automation goals.

Since being deployed at the SNP&SEQ Technology Platform, the Arteria system has been used to process more than 50000 samples and 715 projects, which corresponds to ~1213 Tera-bases of sequencing data.

Clinical Genomics, Uppsala

At the Clinical Genomics Uppsala (Science for Life Laboratory) MPS analyses are developed and implemented for clinical use with the aim to improve diagnostics and allow for targeted treatments. The turnaround time for certain samples must be short, preferably one to two hours from when the facility obtains raw data to when processed data is sent to clinical staff for manual interpretation and reporting.

Raw sequence data is produced at the Uppsala University Hospital, but at present the hospital lacks the capacity to analyse MPS data within the required time. To overcome this, the bioinformatic processing is performed on a private cluster with 8 nodes at Uppsala University. Because of this setup our analyses include transfers between networks, including the more secure hospital network that only allows for communication to be initiated from within the hospital.

Our Arteria implementation is deployed in a docker environment, and consists of Arteria and in-house developed workflows. It is routinely used to push data from the hospital to our cluster located in the Uppsala University Network. When raw data is transferred to the cluster, Arteria subsequently initiates analyses on the cluster, pulls back results into the hospital network and archives data. We also use Arteria to send out emails when results are available or when an error occurs in the sample processing.

The need for a minimal turnaround time and our network limitations makes Arteria and its micro-services a key component for us. The solution automates several tasks that previously were done manually, reducing labour-intensive and time consuming repetitive steps in our pipelines. After being deployed, the system has processed 2176 samples originating from 362 sequence runs. Apart from automating a repetitive task, we estimate that we now save ~2 hours of bioinformatician working time for each sequencing run and slightly improved our turnaround time.

We are currently working on implementing the system on remaining pipelines that we have set up. In the near future we will also implement a solution for the automatic conversion of raw sequence data from sequencing machines to the standard FASTQ format, using the arteria-bcl2fastq micro-service.

The University of Melbourne Center for Cancer Research (UMCCR)

The University of Melbourne Center for Cancer Research (UMCCR) aims to improve cancer patient outcome and enable personalized medicine through rapid whole genome (WGS) and transcriptome (WTS) sequencing of tumor samples. Operating in a clinical, accredited environment requires reproducible and traceable data management which the Center implements through Arteria, providing crucial automation, error notifications, provenance, LIMS control, centralized monitoring and orchestration.

Rapid WGS/WTS data is generated by Illumina's NovaSeq platform and processed through the bcbio framework [21] both at commercial cloud provider (Amazon Web Service) and traditional high-performance computing (HPC) centers within Australia. Arteria automates primary data movement between computational environments and handles distribution of results to a cBio portal, long term archival storage facilities and other downstream services such as automatic report generation through the Personal Cancer Genome Reporter [22]. This software suite includes multi-gigabyte data bundles and multiple deployment steps which have been automated to be installed and configured as new releases become available [23]. The resulting deployed image can be instantiated by StackStorm to process the incoming genomic data deposited on secure cloud storage locations on demand, without idle or wasted CPU cycles, enabling UMCCR to grow as patient numbers increase.

The Arteria solution is deployed to Amazon Web Services, using a CI/CD (Continuous Integration and Continuous Deployment) approach. The entire system is developed and published to GitHub, where incoming changes are automatically tested using the continuous integration system TravisCI. If all tests are successful, new code is deployed into the cloud using the vendor's mechanisms, allowing new changes are brought into production without human intervention. This is illustrated by figure 4.

Utilizing a hybrid approach takes advantage of the high reliability and flexibility provided by commercial IT providers, while still being able to carry out heavy and potentially sensitive computations in an in-house environment. In this environment Arteria offers a modular and reusable framework that eases common integration and middleware issues with systems like LIMS, data management and archival.

Discussion and conclusion

In this paper, we describe the automation system Arteria, which is built on top of the StackStorm automation platform and the Mistral workflow service. Arteria has successfully been adopted by three separate sequencing core facilities, where it forms a crucial part of their infrastructure, thus demonstrating the usefulness of the approach.

Arteria presents an approach to managing the full breadth of the operational aspects surrounding sequencing center operations. It manages *when* as well as *how* certain processes are to be carried out. Through the use of StackStorm as the orchestration engine, we both have a framework for the development of new functionality and a unified user interface for the system operators. The use of workflows at the process level, through Mistral, reduces the need for additional documentation and lowers the risk of human errors. Furthermore, the use of workflows allows for changes to the process to be code reviewed, in accordance with best practices in software development. Finally, the use of micro-services at the execution level has enabled a greater degree of flexibility in the execution model, a clear separation of responsibilities between services, as well as the integration of existing software. Being able to easily integrate existing software into the system has enabled quicker implementation as it lowers the burden of validation for e.g. the ISO/IEC 17025 standard accreditation.

Arteria takes advantage of existing open source tools and aims at creating an avenue for collaboration between sequencing core facilities. We believe that decoupling process from execution, especially the micro-services developed within the Arteria project, could serve as fertile ground for collaboration. The stand-alone nature of the micro-services means that it should be possible for anyone interested to pick them up and include them in their own operations. Prior to beginning work on Arteria in 2015 we carried out an informal survey of possible systems build upon. The main contender to StackStorm at the time appeared to be Airflow [13]. However, we judged that StackStorm had better documentation, which would make it a better choice of platform for us.

One important aspect when adopting novel software is whether the software can be expected to be maintained over time. While there is currently no explicit funding for the Arteria project , it’s already being used across multiple sequencing core facilities. This means that there is already an existing community that can be approached for support. Furthermore, by building Arteria on larger and company-backed projects (i.e. Stackstorm and Mistral), the maintenance burden for most of the underlying functionality is deferred to those projects and should mitigate the risk of the Arteria project being abandoned. Finally, the separation of the systems into independent components means that different parts of the system, for example the micro-services, can still be used, even if support for other parts would be discontinued. In fact, the different parts of the system e.g. the workflow engine, are in themselves interchangeable, thus the entire projects does not rest on the continued maintenance of a single component in it.

It should be noted that since the Arteria project does not provide out-of-the-box solutions, but rather demonstrates how facilities can build their own to suite their particular process, adopters of the Arteria system should expect to update their own systems as necessary. This includes StackStorm, workflows and the micro-services being used.

We recognize that this type of approach has a higher initial overhead than, for example, an orchestration system based on scripts and cron-tab entries. This overhead includes additional

hardware requirements (current production hardware requirements are: a quad core CPU, >16GB RAM, 40G of storage) and increased system complexity. The increased complexity means that personnel resources, with experience in Linux systems and software development, must be dedicated to the development and maintenance of an Arteria system. This is particularly true in the initial implementation phase of a new system. We estimate that to implement Arteria at the SNP&SEQ Technology Platform we dedicated two full time-equivalents (FTE) over a period of 1.5 years. This has since decreased, and we estimate that today we dedicated 0.5 FTE in developing and maintaining the system. The exact time spent per month varies widely, from close to 0 FTE in months when we only apply minor upstream updates to StackStorm to 1 FTE in months when we add new functionality.

Considering the costs of both hardware and personnel, a core facility considering implementing an Arteria system (or any similar system) need to weigh the costs versus the benefits of adopting it. In our opinion, important things to consider include:

- the number of sequencing runs that need to be processed in a year.
- the amount of manual work that can be accepted per sequencing run.
- turn-around time requirements (e.g. time to response to a clinician in clinical sequencing applications).
- diversity of processes, e.g. supporting many different sequencing applications that require different processing workflows.
- complexity of workflows, i.e. how many tasks make up a workflow, and if the workflows contain branching, conditionals, etc.
- the need for traceability, i.e. how important it is to be able to log each action taken in the system for future audit.

Considering the above, there are multiple scenarios in which it could be a good idea to adopt an Arteria system, as it may help in dealing with these issues. For example, a sequencing facility having a large number of sequencing runs per year, e.g. one per day, and that have high demands on rapid

turn-around time, may benefit from using this approach. Another example could be that a sequencing facility has few sequencing runs, but many processes, and complex workflows. Also in this scenario, an Arteria system might be a good option. Note that the amount of data is not a primary consideration in this decision. Many small sequencing runs tend to produce more work than few large ones.

There are situations where spending the required resources does not make sense. For example, if you need to process relatively few sequencing runs, e.g. one per week, and all those runs can be processed in a relatively straight forward manner, the additional overhead introduced by adopting an Arteria system, may not be worth the investment.

However, under the right circumstances, we are confident that the additional overhead pays off, by proving a solid and extensible framework for developing new functionality in accordance with a core facility's needs, without requiring extensive changes to the existing infrastructure.

In the future we expect to keep improving on the Arteria system. In particular we aim at improving the micro-services. Two improvements we are planning on are; firstly, the standardization of the micro-services APIs. Secondly, the implementation of https support in the micro-services, to ensure that communication to and from the services are encrypted. We would like to note however, that we still recommend running the micro-services behind a reverse proxy, in order to handle authentication/authorization, and encryption at a central point. Furthermore, we recommend that Arteria is run in a private network (physical or virtual), for further security. A complete discussion on the securing of web applications is, however, out of scope for this paper, and we recommend that any deployment of an Arteria system is secured according to industry standards.

In conclusion, the Arteria system presents a scalable and flexible solution to the operational issues of data management and analysis faced by sequencing core facilities. All components of Arteria are open source and available to the wider community (https://github.com/arteria-project), and the validity of the approach is demonstrated by the fact that multiple centers have Arteria systems handling their

operations. Finally, we hope that the design described here can be instructive for anyone who needs to implement an orchestration system in the context of a sequencing core facility, or elsewhere.

Availability of supporting source code and requirements

Project name: The Arteria project

Project home page: https://arteria-project.github.io/

Operating system(s): Linux

Programming language: Python

Other requirements: Docker, Docker Compose, make

License: MIT

SciCrunch RRID: SCR_017460

The package arteria-packs, which features Docker images for the system described in this paper, is available for download at: https://github.com/arteria-project/arteria-packs.

Availability of Supporting Data

The data set supporting the results of this article, “Reduced size Illumina NovaSeq runfolder”, is available in the Zenodo repository [24]. A snapshot of the code is also available via the *GigaScience* GigaDB repository[25].

Declarations

List of abbreviation

CD	Continuous delivery
CI	Continuous integration
HPC	High-performance computing
LIMS	Laboratory information management systems
MPS	Massively parallel sequencing

Ethics approval and consent to participate

Not applicable

Consent for publication

Not applicable

Availability of data and material

Not applicable.

Competing interests

The authors declare that they have no competing interests.

Funding

This work was supported by the R&D group at the SNP&SEQ Technology Platform in Uppsala. This facility is part of the National Genomics Infrastructure (NGI) Sweden and Science for Life Laboratory. The SNP&SEQ Platform is also supported by the Swedish Research Council and the Knut and Alice Wallenberg Foundation. UMCCR work was funded through the Australian Genomics Health Alliance which is supported by the National Health and Medical Research Council

(GNT1113531). The Clinical Genomics Uppsala is part of the Diagnostics Development platform within Science for Life Laboratory. Work at the Clinical Genomics Uppsala facility was also supported by grants from the Akademiska University Hospital (ALF-717721).

Authors' contributions

JD, JH, SS, and PL conceptualized the system. JD, JH, SS, ML, PS, RVG, and PL contributed source code. All authors have contributed to writing the manuscript. All authors read and approved the final manuscript.

Acknowledgements

The entire bioinformatics team at the SNP&SEQ Technology Platform has contributed to the Arteria project with feedback during its adoption. Especially Monika Brandt, Matilda Åslin and Sara Ekberg have provided extremely useful feedback based on their daily operations of the system, as well as contributing code to the project. Parts of the computations for the Arteria Project were performed on resources provided by SNIC through Uppsala Multidisciplinary Center for Advanced Computational Science (UPPMAX).

We would like to acknowledge Samuel Lampa and Jessica Nordlund for reading this text and providing highly valuable comments, which have been instrumental in developing this manuscript.

Figures

Figure 1 - An overview of the conceptual levels of the Arteria project.

Figure 2 - Description of the StackStorm event model. Sensors will perceive events in the environments, e.g. a file being created or a certain time of day it occurs. This passes information to the rule layer where the data is evaluated and depending on which, if any, criteria are fulfilled one or more actions are triggered. Actions can be single commands or full workflows to be executed.

Figure 3 - Schematic view of a system deployment scenario, showing how data is written to the local storage and compute nodes from the sequencing machines, and how the system uses information and resources from multiple sources to coordinate the process. The operator can then monitor and control the processes from the single interface provided at the master automation node.

Figure 4 - UMCCR arteria cloud infrastructure. When a commit is pushed to our github repository and validated by TravisCI, it proceeds to our autoscaling group "arteria" which subsequently deploys cloud instances, incorporating the new Arteria and StackStorm code changes. After changes are deployed, any incoming event such as a new sequencing run being completed, are handled by this newly deployed code and data is copied from the sequencers to our university HPC center for further downstream processing with bcbio [21].

References

1. Lander ES, Linton LM, Birren B, Nusbaum C, Zody MC, Baldwin J, et al. Initial sequencing and analysis of the human genome. Nature. 2001;409:860–921.

2. Spang A, Saw JH, Jørgensen SL, Zaremba-Niedzwiedzka K, Martijn J, Lind AE, et al. Complex archaea that bridge the gap between prokaryotes and eukaryotes. Nature. 2015;521:173–9.

3. 1000 Genomes Project Consortium, Auton A, Brooks LD, Durbin RM, Garrison EP, Kang HM, et al. A global reference for human genetic variation. Nature. 2015;526:68–74.

4. Ashley EA. Towards precision medicine. Nat Rev Genet. 2016;17:507–22.

5. Goodwin S, McPherson JD, McCombie WR. Coming of age: ten years of next-generation sequencing technologies. Nat Rev Genet. 2016;17:333–51.

6. Stephens ZD, Lee SY, Faghri F, Campbell RH, Zhai C, Efron MJ, et al. Big Data: Astronomical or Genomical? PLoS Biol. 2015;13:e1002195.

7. Spjuth O, Bongcam-Rudloff E, Dahlberg J, Dahlö M, Kallio A, Pireddu L, et al. Recommendations on e-infrastructures for next-generation sequencing. Gigascience. 2016;5:26.

8. Spjuth O, Bongcam-Rudloff E, Hernández GC, Forer L, Giovacchini M, Guimera RV, et al. Experiences with workflows for automating data-intensive bioinformatics. Biol Direct. 2015;10:43.

9. Lampa S, Alvarsson J, Spjuth O. Towards agile large-scale predictive modelling in drug discovery with flow-based programming design principles. J Cheminform. 2016;8:67.

10. Leipzig J. A review of bioinformatic pipeline frameworks. Brief Bioinform. 2017;18:530–6.

11. Amstutz P, Crusoe MR, Tijanić N, Chapman B, Chilton J, Heuer M, et al. Common Workflow Language, v1.0. Figshare. 2016. doi:10.6084/m9.figshare.3115156.v2.

12. Spotify. Luigi. GitHub. 2017. https://github.com/spotify/luigi. Accessed 24 Jan 2017.

13. Apache. https://github.com/apache/incubator-airflow. GitHub. 2017. https://github.com/apache/incubator-airflow. Accessed 24 Jan 2017.

14. Cuccuru G, Leo S, Lianas L, Muggiri M, Pinna A, Pireddu L, et al. An automated infrastructure to support high-throughput bioinformatics. In: 2014 International Conference on High Performance Computing & Simulation (HPCS). IEEE; 2014. p. 600–7.

15. Afgan E, Baker D, van den Beek M, Blankenberg D, Bouvier D, Čech M, et al. The Galaxy platform for accessible, reproducible and collaborative biomedical analyses: 2016 update. Nucleic Acids Res. 2016;44:W3–10.

16. RabbitMQ - Messaging that just works. http://www.rabbitmq.com/. Accessed 7 Feb 2018.

17. StackStorm. StackStorm/st2. GitHub. 2017. https://github.com/StackStorm/st2. Accessed 24 Jan 2017.

18. Mistral. 2016. https://wiki.openstack.org/wiki/Mistral. Accessed 16 Aug 2016.

19. ISO/IEC 17025:2005 - General requirements for the competence of testing and calibration laboratories. 2014. https://www.iso.org/standard/39883.html. Accessed 10 Apr 2017.

20. Illumina. bcl2fastq2 Conversion Software v2.17. http://support.illumina.com/downloads/bcl2fastq-conversion-software-v217.html. Accessed 15 Aug 2016.

21. Chapman B. bcbio-nextgen. https://github.com/chapmanb/bcbio-nextgen. Accessed 8 Feb 2018.

22. Nakken S, Fournous G, Vodák D, Aasheim LB, Myklebost O, Hovig E. Personal Cancer Genome Reporter: variant interpretation report for precision oncology. Bioinformatics. 2017. doi:10.1093/bioinformatics/btx817.

23. Personal Cancer Genome Reporter deployment recipes. https://github.com/umccr/pcgr-deploy. Accessed 8 Feb 2018.

24. Dahlberg, Johan, Larsson, Pontus, & Liljedahl, Ulrika. (2018). Reduced size Illumina NovaSeq runfolder (Version 1.0.0) [Data set]. Zenodo. http://doi.org/10.5281/zenodo.1204292

25. Dahlberg J; Hermansson J; Sturlaugsson S; Lysenkova M; Smeds P; Ladenvall C; Guimera RV; Reisinger F; Hofmann O; Larsson P (2019): Supporting data for "Arteria: An automation system for a sequencing core facility" GigaScience Database. http://dx.doi.org/10.5524/100666

figure 1

Click here to access/download;Figure;figure1.pdf

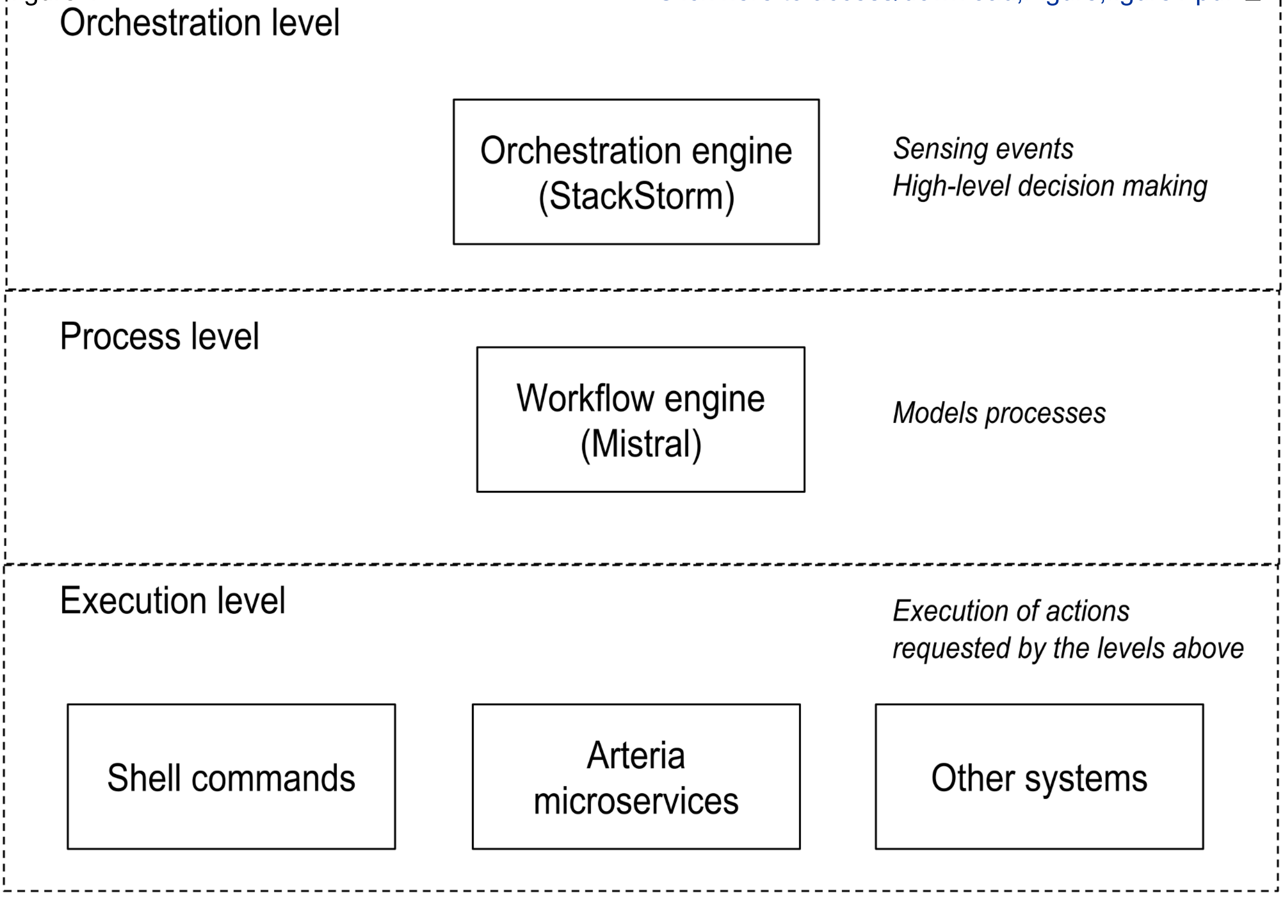

figure 2

Click here to access/download;Figure;figure2.pdf

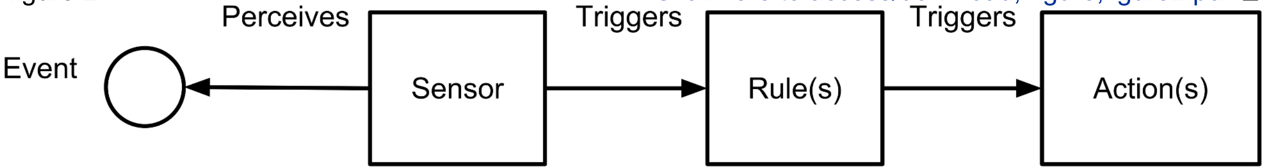

figure 3 Click here to access/download;Figure;figure3.pdf

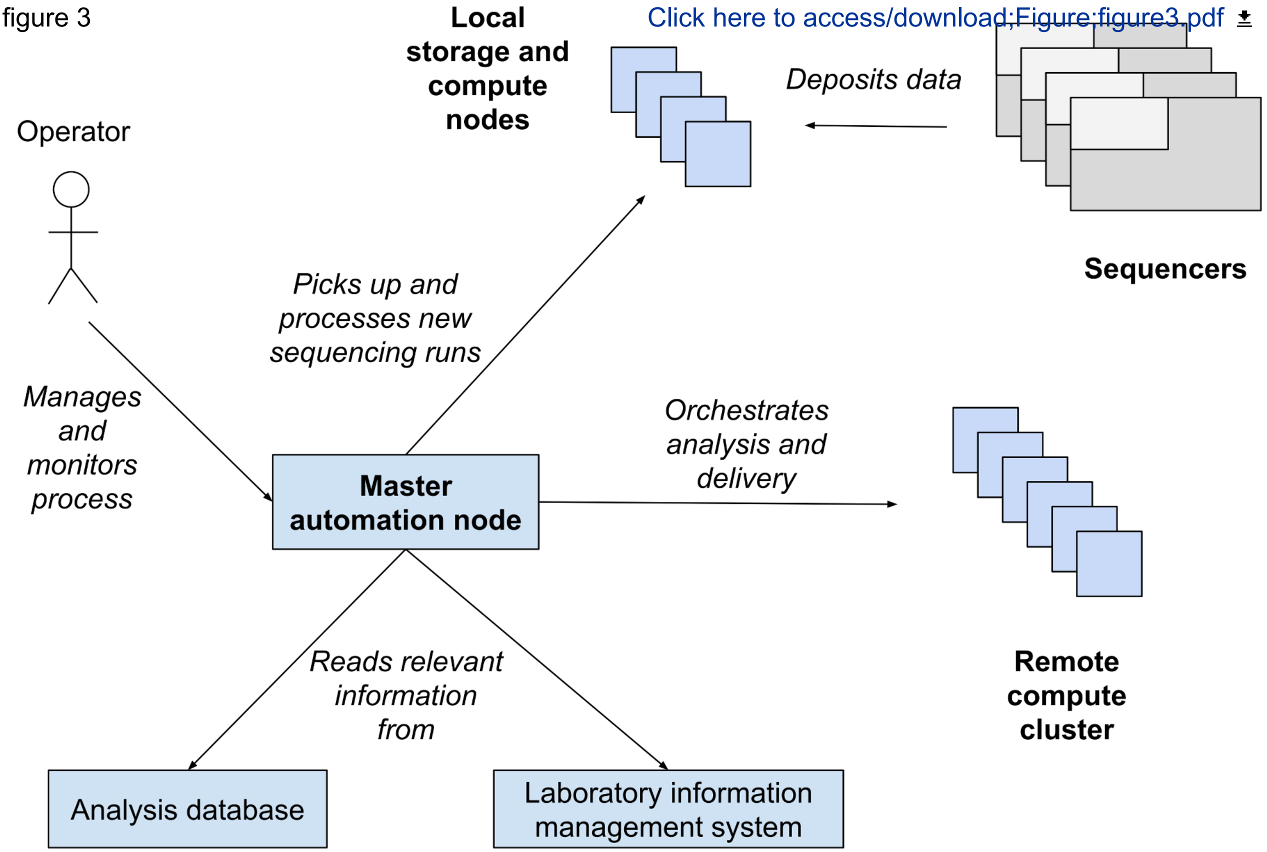

figure 4

Click here to access/download;Figure;figure4.pdf

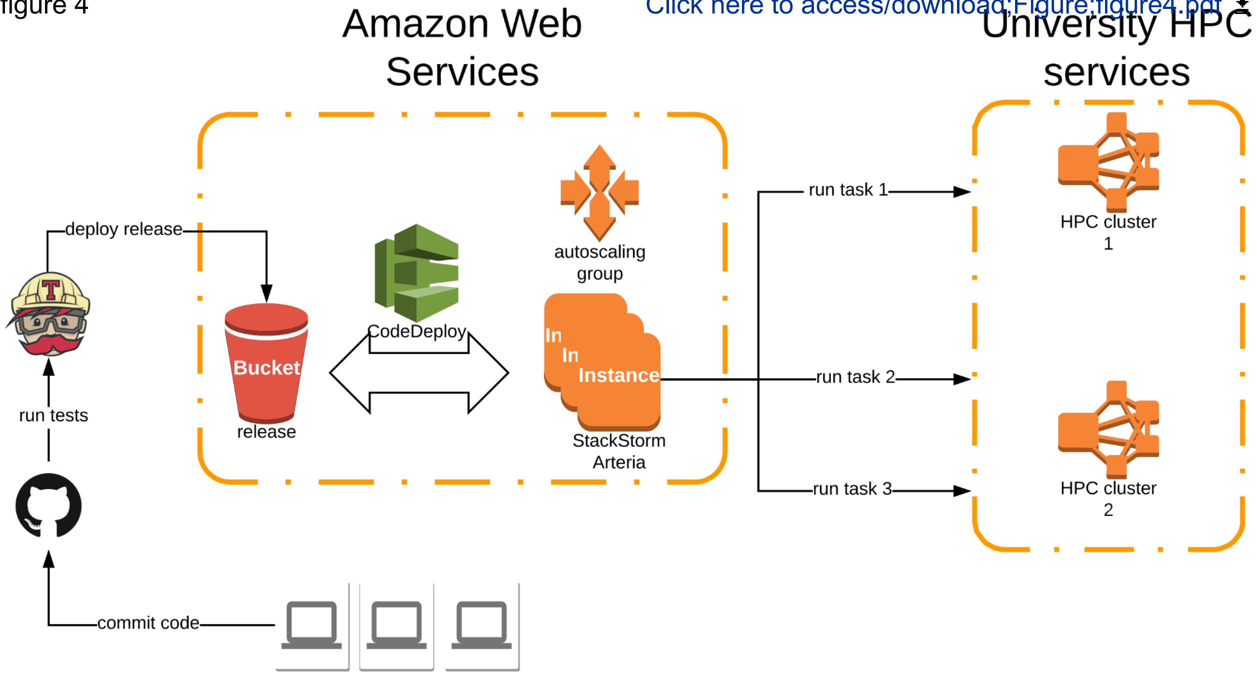

Supplementary figure 1

Click here to access/download
Supplementary Material
example_arteria_workflow.png
