# Supplementary figures and images for: Arteria: An automation system for a sequencing core facility
Source: Gigascience. 2019 Dec 11;8(12):giz135. doi: 10.1093/gigascience/giz135 (PMC6905352; doi:10.1093/gigascience/giz135)

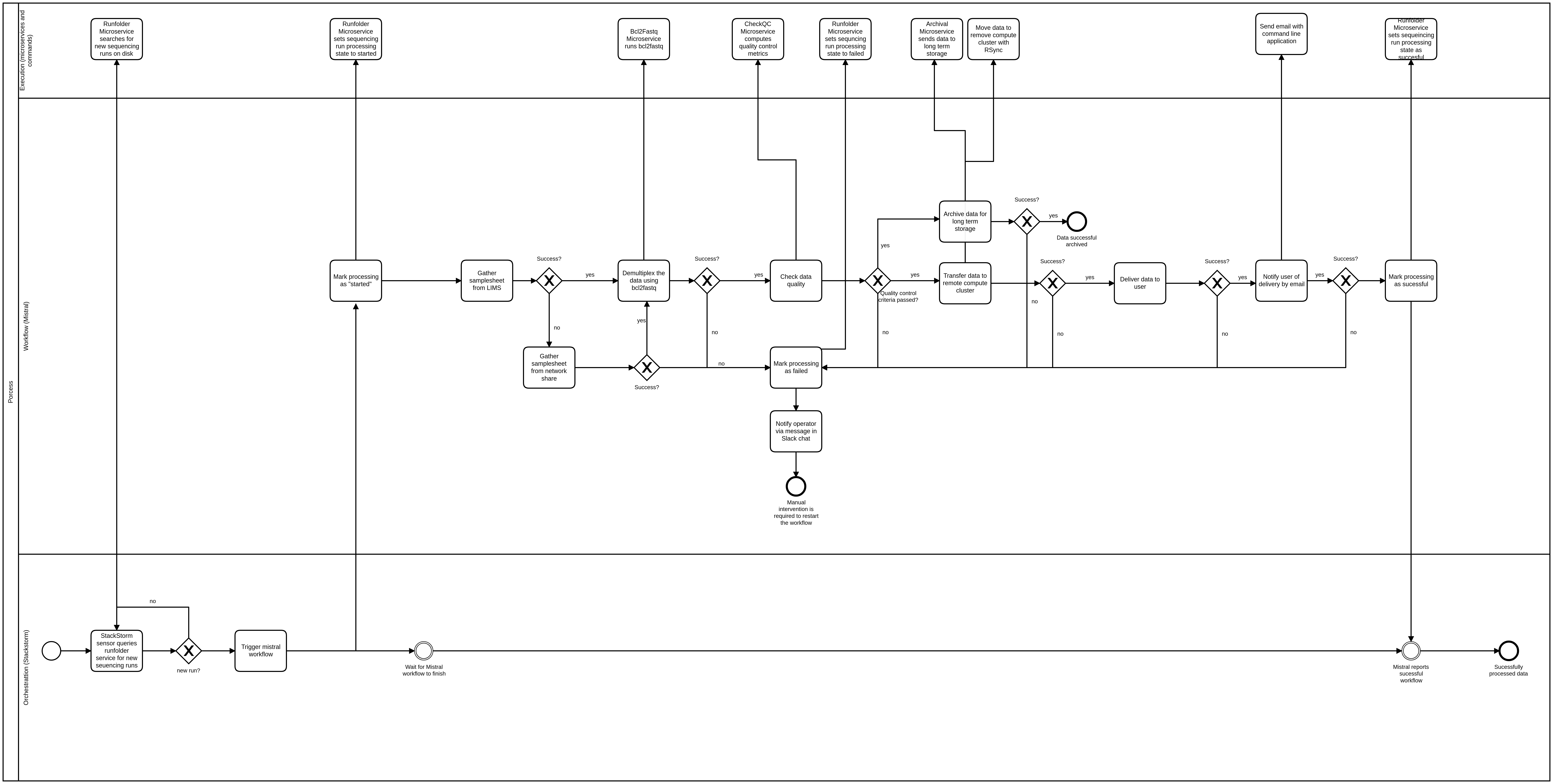

Supplement: giz135_Supplemental_Figure [file giz135_supplemental_figure.png]
