# Supplementary material for: Arteria: An automation system for a sequencing core facility
Source: Gigascience. 2019 Dec 11;8(12):giz135. doi: 10.1093/gigascience/giz135 (PMC6905352; doi:10.1093/gigascience/giz135)
Supplement: giz135_Response_to_Reviewer_Comments_Original_Submission [file giz135_response_to_reviewer_comments_original_submission.pdf]

Author's Response To Reviewer Comments

Close

Dear editor and reviews,

Thank you for your insightful comments. They have really helped in improving the manuscript. We have made changes to the manuscript in accordance with your suggestions. In order to make it easier to follow how we have addressed your suggestions, we have written answers after each paragraph, surrounded by “>>>”.

Once again, many thanks for the excellent feedback, and we hope that you feel that the changes we have made are to your satisfaction.

Yours sincerely,
Johan Dahlberg, PhD

Reviewer #1
The authors present a computational framework built over existing open-source technologies like the StackStorm, to develop an event-driven automation platform for processing sequencing data. Automation and workflow management still represents a significant challenge on many Sequencing facilities, there here presented system is a step in the right direction and should capture the attention of the community.

Other workflow management systems such as snakemake and nextflow are available, the community how these systems compare with the here presented framework? for instance,
the system presented here uses the Python ecosystem. The Python ecosystem is a mess to work with when it comes to 3rd party libraries that need to be installed on HPC. The installation often requires environment management, not all of which are solvable with virtualenv's or conda. Nextflow installs seamlessly on any system that has Java 8.

>>>
We do not explicitly compare Arteria, and in particular Mistral, to other workflow system, in the article because the problem we are trying to solve is a slightly different one. Snakemake and Nextflow are focused on running bioinformatical tools. Typically command line tools which have files are their inputs and outputs. Consequently, they are well suited to solving that type of problem. Arteria focuses instead of solving issues of operational automation, e.g. moving data between compute clusters, and updating databases. A step in that process may be to use e.g. Nextflow or Snakemake to carry out more complex analysis workflows. So, as part of an Arteria workflow, one might trigger a Nextflow workflow to be run, and then fetch the files and upload them to a file server for long term storage.

We have tried to clarify this distinction in the “Challenges in, and approaches to, processing sequencing data” section now.

When it comes to the Python ecosystem, we acknowledge the points that you bring up. However, we believe that any programing language comes with its own set of pros and cons. In this case we have decided to build upon Python mainly for two reasons. Firstly, Python is the language used by the native StackStorm APIs, so deviating from the would be inconvenient. Secondly, Python is a language with a high adoption rate in the bioinformatics community, which means that it should be easier for others to understand and modify our code.
>>>

Besides what a tool can or cannot do, potential users need to check the quality of the documentation, whether it is actively developed and maintained, how many developers contribute to it, and size of the user base. The authors clearly describe two case-studies at the SNP&SEQ Technology Platform sequencing core facility at Science for Life Laboratory and the Clinical Genomics, Uppsala, however, it will be important to know what is the plan for continued funding, development, and maintenance for this

system, this is very important in order to make it an attractive and sustainable alternative for the community.

>>>
This is a good point. Arteria, like many projects of this type, lack dedicated funding. At the SNP&SEQ Technology Platform we have deeply invested in it, and base our entire operations on it. However, we recognize that this is not the same as having a guaranteed that the project will be maintained indefinitely.

We hope that by basing Arteria on an existing framework which is backed by a much larger community, some of the risk of the project becoming orphaned is mitigated. As long as StackStorm is maintained, the orchestration and workflow levels of the systems should get updates, while it would be incumbent on users of the system to provide updates to the microservices they use.

We have added a paragraph in the discussion that deals with this point now.
>>>

A case study is presented to demonstrate the system's usability. Illumina bcl2fastq tools is used to perform the demultiplexing (dividing sequence reads into separate files for each index tag/sample) and generating the fastq data files required for downstream analysis, for some Illumina sequencing platforms, this step is carried out automatically using the onboard PC. For others, this step is just a simple Linux command line. In order to really demonstrate the workflow management abilities of this platform, the authors should incorporate other downstream analysis steps like raw data quality control with FASTQC, mapping, feature quantification (for RNA-Seq) or Variant Calling (for DNA-seq) in their demo/example case study.

>>>
As discussed above, the purpose of Arteria is focused on operational rather than analytical problems. We hope that the explanation and accompanying changes described above have adequately addressed your concerns on this topic. We have added a picture to the supplementary materials which exemplifies the type of workflows that Arteria is focused on.
>>>

How the system handles the necessary user-defined parameters for a particular task? for instance, the bcl2fastq process usually needs a sample sheet - a simple comma separated file (csv) with the library chemistry, sample names and the index tag used for each sample, in addition to some other metrics describing the run, this will, of course, needs to be customized for different users, per-run or per project. In a similar manner, the incorporation of further downstream analysis steps on the pipeline will require a user-defined sample description table (i.e. for DEG detection).

>>>
We have added a clarification to the “Event-based orchestration” section, to make it explicit that when starting an action (e.g. a workflow) manually, the user may (and sometimes must) provide parameters.
>>>

How the system handles conditional creation of events based on the input data? for instance, Snakemake allows for conditional creation of the DAG and conditional execution of different code based on the input. Is this feature supported by the system?

>>>
Yes, this feature is supported by the system. We describe it in the section “Modelling processes as workflows”:

“It supports the use of conditionals, forking (defining multiple tasks that must be run after the completion of a given task) and joining (the synchronization of multiple parallel workflow branches and aggregation of their data).”
>>>

Does the system feature singularity support with the singularity directive? This is an important feature since not all potential HPC users will have root access to deploy Docker containers in their infrastructures.

>>>
No. The system does not have direct support for singularity, and is aimed at users, which at least in part control their own infrastructure, and can install software, open ports in firewalls, etc. However, that said, services can be run through singularity using the service concept (https://sylabs.io/guides/3.0/user-guide/running_services.html). We view singularity, like docker, or other container technologies as complementary to the micro-services, not as something that can replace them.
>>>

Reviewer #2
The authors describe the Arteria system for sequencing core automation. Arteria is a mechanism for fully automating the analysis parts of a sequencing core, including fastq generation and QC, data transfer/archiving, and data removal, and more generally for thinking about operational aspects of a sequencing core in a structured, site-agnostic way. Arteria is based on other open source technologies: StackStorm for orchestration and Mistral workflow language. The authors argue that by depending on these external packages, they are free to concentrate on the sequencing specific requirements. As a result, Arteria is not a self-contained piece of software. Instead it is all of the 'glue code' required to use StackStorm and Mistral for the purposes of a sequencing centre, as well as the specific microservices that are REST interfaces for launching processes like bcl2fastq.

This is important work that has been little-spoken-of in the bioinformatics analysis community and I think that Arteria contributes greatly to the discussion and ongoing improvement. The concepts, separation of concerns, and focus on good, secure design are fundamental to the way we think about sequencing core automation. Relying on open source software is a good idea to reduce the amount of overhead and reliance on individual sequencing centres. The flexibility of the system to adapt to new centres is exemplified by the three separate use-cases. I was quite impressed by the ability to run a CU/CD approach for one of the use-cases.

It would be interesting and contribute to overall understanding of the system to have a figure or text description of a specific process from end to end, e.g. what kicks off when the 'sequencing is done' sensor is triggered. There is a very short description, but it would be interesting to see the system process diagram of when StackStorm and Mistral are contacted with what information, when the services launched contact other services, where reports/emails are generated, etc. All of this is essentially already in the Github project, in a less friendly way. It would be especially interesting if you included details such as what happens when something goes wrong, for example, if the LIMS has the incorrect molecular index and bcl2fastq fails.

>>>
We have added a diagram showing how information flows between different levels in the system now. This is however, rather large, so we have opted to add it as a supplementary figure rather than to add it into the manuscript proper.
>>>

The discussion section feels thin, with little to no comparison of their method to other methods. Some of what I expected here has been included in the 'Findings' section, but the discussion should be an opportunity to honestly examine what has been done in context with other methods. The authors do mention that there isn't much published on this topic, but a short examination of the benefits of this method compared to other workflow engines, other event schedulers, other bioinformatics methods would be appreciated. A few suggestions are included below.

>>>
Thank you for providing excellent feedback on the discussion. We will address the different suggestions below, to make it easier to follow up on.
>>>

Who is this system for? Should centres need 2 Novaseqs before they consider Arteria? What is the minimum size of operation that would make this infeasible? On the other end, how much can these systems really scale?

>>>
We have added a section to the discussion, that elaborates on what we think are important things to consider when deciding whether to adopt an Arteria system or not. Naturally, it is difficult to provide a hard boundary for when we would adopt recommendation since there are so many factors to consider.

However, we hope that you find the points of discussion useful.
>>>

Automation systems are rarely published because every core requires small variation in procedure, infrastructure, surrounding systems. Each use case mentions how much time is saved, but how much effort is it for an institution to set up these systems? An estimate with number of people and months/years of effort would be sufficient.

>>>
We have included estimates of how much time we have dedicated to the development and maintenance of the system at the SNP&SEQ Technology platform, both in the initial, implementation phase, as well as now that we are mainly maintaining the system.
>>>

The authors do not provide any justification for why they are using StackStorm and Mistral in particular. What benefits do these services offer compared to other orchestration and workflow engines? What are the main competitors?

>>>
Prior to starting work on Arteria in 2015 we did an informal survey of systems that we thought could fulfill the needs that we saw. One feature in particular that made StackStorm stand out was the use of sensors to detect events in the environment - while there are many workflow systems, from or brief survey, few seemed to support this way of automatically starting workflows. The main competitor at the time was Airflow, but at that time, at least the StackStorm documentation was superior to the Airflow documentation, and that made us select StackStorm. We have added a paragraph on this in the discussion now.
>>>

One thing I am always concerned about when it comes to using other software packages is whether it will be supported long term, and what happens if or when support is removed for it. How entangled are Arteria's systems with StackStorm and Mistral? What happens when one or both of them are updated?

>>>
Concerning long term support, Arteria, like many projects of this type, lack dedicated funding. At the SNP&SEQ Technology Platform we have deeply invested in it, and base our entire operations on it. However, we recognize that this is not the same as having a guaranteed that the project will be maintained indefinitely.

We hope that by basing Arteria on an existing framework which is backed by a much larger community, some of the risk of the project becoming orphaned is mitigated. As long as StackStorm is maintained, the orchestration and workflow levels of the systems should get updates, while it would be incumbent on users of the system to provide updates to the microservices they use.

The separation between levels in the Arteria model, should further mitigate the risk. For example, one can still use the micro-services without using Mistral as a workflow engine, and one could switch out the workflow engine used, without making changes to the micro-service, etc.

We have added a paragraph in the discussion that deals with this point now.
>>>

What future work is expected on the system? Is there any maintenance expected, for example when there is new version of Mistral, or is each sequencing site on their own?

>>>
We expect to maintain the example implementation (github.com/arteria-project/arteria-packs), as well as the micro-services associated with the project. However, since most sequencing facilities will implement their own custom workflows, each site will have to update StackStorm, Mistral, etc according to their own needs. We have added a paragraph to the discussion to clarify that.
>>>

About the Arteria microservices. There are several implemented microservices based on Tornado, a python package for implementing web services. These seem to have a bespoke shape, i.e. each

microservice is different from the next. With everything else so defined, I found this an interesting oversight. I would like a short discussion of what kind of information a microservice needs provided. Have the authors considered any of the interfaces from the Global Alliance for Global Health (GA4GH)? In particular, the workflow execution schema or task execution schema? Was there any consideration of using Docker or other container technology instead of microservices?

>>>
We completely agree that standardization of the APIs of the micro-services is an important area where we could improve in the future. We have looked at the specifications provided by the GA4GH, however, we are not convinced that any of the Task Execution Service API or the Workflow Execution Service API, are a perfect match. The former focuses on abstracting the submission of tasks to e.g. a cluster scheduler, and the latter on running workflows based on e.g. Common Workflow Language. We can see a scenario in which the service in turn communicates with a service implementing either API, but the services themselves are meant to abstract away many of the details that are required from those APIs.

We consider container technologies to be complementary to the use of micro-service. For example, in the example implementation of an Arteria, we run the micro-services in docker containers which are orchestrated by docker compose.

We have added a section to the discussion, about this.
>>>

The execution-level microservices are implemented on HTTP (unencrypted) microservices. Especially since several of the use-cases involve not only the analysis, but also the transfer of clinical human data, there should be a small note about securing these systems against unauthorized access and interception. What architecture is necessary in order to keep these secure? What should a new site absolutely not do?

>>>
Adding https support to the micro-services is something that is on our roadmap. However, we recommend using a reverse-proxy to handle encryption, authentication/authorization. In our internal setup we use Kong for this purpose. In general we do not recommend running Arteria in open networks, but rather recommend that it is run in a section of private network (physical or virtual). We have added a section to the discussion on this topic, however, we do feel that a complete discussion on the security of web-applications is out of scope for this paper.
>>>

All in all, this paper is a great contribution to this discussion. As the authors say, not much has been published in this domain before and that's an enormous oversight. Hopefully this paper can begin the discussion around such automation systems. I absolutely support publication after addressing a few of the points above.

Close
