# Supplementary material for: Arteria: An automation system for a sequencing core facility
Source: Gigascience. 2019 Dec 11;8(12):giz135. doi: 10.1093/gigascience/giz135 (PMC6905352; doi:10.1093/gigascience/giz135)
Supplement: giz135_Reviewer_1_Report_Original_Submission — Jorge Andrade, Ph.D. -- 6/20/2019 Reviewed [file giz135_reviewer_1_report_original_submission.pdf]

Reviewer Report

Title: Arteria: An automation system for a sequencing core facility

Version: Original Submission **Date:** 6/20/2019

Reviewer name: Jorge Andrade, Ph.D.

Reviewer Comments to Author:

The authors present a computational framework built over existing open-source technologies like the StackStorm, to develop an event-driven automation platform for processing sequencing data. Automation and workflow management still represents a significant challenge on many Sequencing facilities, there here presented system is a step in the right direction and should capture the attention of the community.

Other workflow management systems such as snakemake and nextflow are available, the community how these systems compare with the here presented framework? for instance,

the system presented here uses the Python ecosystem. The Python ecosystem is a mess to work with when it comes to 3rd party libraries that need to be installed on HPC. The installation often requires environment management, not all of which are solvable with virtualenv's or conda. Nextflow installs seamlessly on any system that has Java 8.

Besides what a tool can or cannot do, potential users need to check the quality of the documentation, whether it is actively developed and maintained, how many developers contribute to it, and size of the user base. The authors clearly describe two case-studies at the SNP&SEQ Technology Platform sequencing core facility at Science for Life Laboratory and the Clinical Genomics, Uppsala, however, it will be important to know what is the plan for continued funding, development, and maintenance for this system, this is very important in order to make it an attractive and sustainable alternative for the community.

A case study is presented to demonstrate the system's usability. Illumina bcl2fastq tools is used to perform the demultiplexing (dividing sequence reads into separate files for each index tag/sample) and generating the fastq data files required for downstream analysis, for some Illumina sequencing platforms, this step is carried out automatically using the onboard PC. For others, this step is just a simple Linux command line. In order to really demonstrate the workflow management abilities of this platform, the authors should incorporate other downstream analysis steps like raw data quality control with FASTQC, mapping, feature quantification (for RNA-Seq) or Variant Calling (for DNA-seq) in their demo/example case study.

How the system handles the necessary user-defined parameters for a particular task? for instance, the bcl2fastq process usually needs a sample sheet - a simple comma separated file (csv) with the library chemistry, sample names and the index tag used for each sample, in addition to some other metrics describing the run, this will, of course, needs to be customized for different users, per-run or per project. In a similar manner, the incorporation of further downstream analysis steps on the pipeline will require a user-defined sample description table (i.e. for DEG detection).

How the system handles conditional creation of events based on the input data? for instance,

Snakemake allows for conditional creation of the DAG and conditional execution of different code based on the input. Is this feature supported by the system?
Does the system feature singularity support with the singularity directive? This is an important feature since not all potential HPC users will have root access to deploy Docker containers in their infrastructures.

Level of Interest

Please indicate how interesting you found the manuscript: Choose an item.

Quality of Written English

Please indicate the quality of language in the manuscript: Choose an item.

Declaration of Competing Interests

Please complete a declaration of competing interests, considering the following questions:

- Have you in the past five years received reimbursements, fees, funding, or salary from an organisation that may in any way gain or lose financially from the publication of this manuscript, either now or in the future?
- Do you hold any stocks or shares in an organisation that may in any way gain or lose financially from the publication of this manuscript, either now or in the future?
- Do you hold or are you currently applying for any patents relating to the content of the manuscript?
- Have you received reimbursements, fees, funding, or salary from an organization that holds or has applied for patents relating to the content of the manuscript?
- Do you have any other financial competing interests?
- Do you have any non-financial competing interests in relation to this paper?

If you can answer no to all of the above, write 'I declare that I have no competing interests' below. If your reply is yes to any, please give details below.

I declare that I have no competing interests

I agree to the open peer review policy of the journal. I understand that my name will be included on my report to the authors and, if the manuscript is accepted for publication, my named report including any attachments I upload will be posted on the website along with the authors' responses. I agree for my report to be made available under an Open Access Creative Commons CC-BY license (http://creativecommons.org/licenses/by/4.0/). I understand that any comments which I do not wish to be included in my named report can be included as confidential comments to the editors, which will not be published.

Choose an item.

To further support our reviewers, we have joined with Publons, where you can gain additional credit to further highlight your hard work (see: https://publons.com/journal/530/gigascience). On publication of this paper, your review will be automatically added to Publons, you can then choose whether or not to claim your Publons credit. I understand this statement.

Yes Choose an item.
