# Supplementary material for: Arteria: An automation system for a sequencing core facility
Source: Gigascience. 2019 Dec 11;8(12):giz135. doi: 10.1093/gigascience/giz135 (PMC6905352; doi:10.1093/gigascience/giz135)
Supplement: giz135_Reviewer_2_Report_Original_Submission — Morgan Taschuk -- 7/29/2019 Reviewed [file giz135_reviewer_2_report_original_submission.pdf]

Reviewer Report

Title: Arteria: An automation system for a sequencing core facility

Version: Original Submission **Date:** 7/29/2019

Reviewer name: Morgan Taschuk

Reviewer Comments to Author:

The authors describe the Arteria system for sequencing core automation. Arteria is a mechanism for fully automating the analysis parts of a sequencing core, including fastq generation and QC, data transfer/archiving, and data removal, and more generally for thinking about operational aspects of a sequencing core in a structured, site-agnostic way. Arteria is based on other open source technologies: StackStorm for orchestration and Mistral workflow language. The authors argue that by depending on these external packages, they are free to concentrate on the sequencing specific requirements. As a result, Arteria is not a self-contained piece of software. Instead it is all of the 'glue code' required to use StackStorm and Mistral for the purposes of a sequencing centre, as well as the specific microservices that are REST interfaces for launching processes like bcl2fastq.

This is important work that has been little-spoken-of in the bioinformatics analysis community and I think that Arteria contributes greatly to the discussion and ongoing improvement. The concepts, separation of concerns, and focus on good, secure design are fundamental to the way we think about sequencing core automation. Relying on open source software is a good idea to reduce the amount of overhead and reliance on individual sequencing centres. The flexibility of the system to adapt to new centres is exemplified by the three separate use-cases. I was quite impressed by the ability to run a CU/CD approach for one of the use-cases.

It would be interesting and contribute to overall understanding of the system to have a figure or text description of a specific process from end to end, e.g. what kicks off when the 'sequencing is done' sensor is triggered. There is a very short description, but it would be interesting to see the system process diagram of when StackStorm and Mistral are contacted with what information, when the services launched contact other services, where reports/emails are generated, etc. All of this is essentially already in the Github project, in a less friendly way. It would be especially interesting if you included details such as what happens when something goes wrong, for example, if the LIMS has the incorrect molecular index and bcl2fastq fails.

The discussion section feels thin, with little to no comparison of their method to other methods. Some of what I expected here has been included in the 'Findings' section, but the discussion should be an opportunity to honestly examine what has been done in context with other methods. The authors do mention that there isn't much published on this topic, but a short examination of the benefits of this method compared to other workflow engines, other event schedulers, other bioinformatics methods would be appreciated. A few suggestions are included below.

Who is this system for? Should centres need 2 Novaseqs before they consider Arteria? What is the minimum size of operation that would make this infeasible? On the other end, how much can these systems really scale?

Automation systems are rarely published because every core requires small variation in procedure, infrastructure, surrounding systems. Each use case mentions how much time is saved, but how much effort is it for an institution to set up these systems? An estimate with number of people and months/years of effort would be sufficient.

The authors do not provide any justification for why they are using StackStorm and Mistral in particular. What benefits do these services offer compared to other orchestration and workflow engines? What are the main competitors? One thing I am always concerned about when it comes to using other software packages is whether it will be supported long term, and what happens if or when support is removed for it. How entangled are Arteria's systems with StackStorm and Mistral? What happens when one or both of them are updated?

What future work is expected on the system? Is there any maintenance expected, for example when there is new version of Mistral, or is each sequencing site on their own?

About the Arteria microservices. There are several implemented microservices based on Tornado, a python package for implementing web services. These seem to have a bespoke shape, i.e. each microservice is different from the next. With everything else so defined, I found this an interesting oversight. I would like a short discussion of what kind of information a microservice needs provided. Have the authors considered any of the interfaces from the Global Alliance for Global Health (GA4GH)? In particular, the workflow execution schema or task execution schema? Was there any consideration of using Docker or other container technology instead of microservices?

The execution-level microservices are implemented on HTTP (unencrypted) microservices. Especially since several of the use-cases involve not only the analysis, but also the transfer of clinical human data, there should be a small note about securing these systems against unauthorized access and interception. What architecture is necessary in order to keep these secure? What should a new site absolutely not do?

All in all, this paper is a great contribution to this discussion. As the authors say, not much has been published in this domain before and that's an enormous oversight. Hopefully this paper can begin the discussion around such automation systems. I absolutely support publication after addressing a few of the points above.

Level of Interest

Please indicate how interesting you found the manuscript: Choose an item.

Quality of Written English

Please indicate the quality of language in the manuscript: Choose an item.

Declaration of Competing Interests

Please complete a declaration of competing interests, considering the following questions:

- Have you in the past five years received reimbursements, fees, funding, or salary from an organisation that may in any way gain or lose financially from the publication of this manuscript, either now or in the future?
- Do you hold any stocks or shares in an organisation that may in any way gain or lose financially from the publication of this manuscript, either now or in the future?
- Do you hold or are you currently applying for any patents relating to the content of the manuscript?
- Have you received reimbursements, fees, funding, or salary from an organization that holds or has applied for patents relating to the content of the manuscript?
- Do you have any other financial competing interests?
- Do you have any non-financial competing interests in relation to this paper?

If you can answer no to all of the above, write 'I declare that I have no competing interests' below. If your reply is yes to any, please give details below.

I work for a sequencing centre and we have our own open source automation system. I receive no financial compensation for this other than continued employment.

I agree to the open peer review policy of the journal. I understand that my name will be included on my report to the authors and, if the manuscript is accepted for publication, my named report including any attachments I upload will be posted on the website along with the authors' responses. I agree for my report to be made available under an Open Access Creative Commons CC-BY license (http://creativecommons.org/licenses/by/4.0/). I understand that any comments which I do not wish to be included in my named report can be included as confidential comments to the editors, which will not be published.

Choose an item.

To further support our reviewers, we have joined with Publons, where you can gain additional credit to further highlight your hard work (see: https://publons.com/journal/530/gigascience). On publication of this paper, your review will be automatically added to Publons, you can then choose whether or not to claim your Publons credit. I understand this statement.

Yes Choose an item.
